# An endogenous retinoic acid response element enhancer controls *Cyp26a1*-mediated retinoic acid clearance during embryonic stem cell differentiation

**DOI:** 10.1016/j.jbc.2026.113613

**Published:** 2026-09-29

**Authors:** Dongqing Li, Pengpeng Guan, Bo Wang, Wange Lu, Guangsong Su

**Affiliations:** 1 Department of Laboratory Medicine and Institute of Precision Medicine, The First Affiliated Hospital, Sun Yat-sen University, Guangzhou, China; 2 State Key Laboratory of Medicinal Chemical Biology and College of Life Sciences, Nankai University, Tianjin, China

**Keywords:** RA signaling, enhancer, *Cyp26a1*, embryonic stem cells, RARE

## Abstract

Precise control of retinoic acid (RA) signaling is essential for embryonic development, as fluctuations in RA availability can perturb cell fate determination and contribute to severe developmental abnormalities. Yet how inducible RA clearance is encoded within endogenous chromatin during pluripotent cell differentiation remains poorly understood. Here, using RA-induced differentiation of mouse embryonic stem cells (mESCs), we identify *Cyp26a1* as one of the most strongly induced genes during this fate transition. Among genes involved in RA synthesis and metabolism, *Cyp26a1* is distinguished by the formation of a highly RA-responsive distal enhancer. This enhancer contains functional RA response elements (RAREs) and is activated by ligand-dependent RAR/RXR binding, p300 recruitment, and H3K27ac deposition, accompanied by increased enhancer-promoter interaction. Genetic deletion of the enhancer or its core RARE motifs markedly impairs *Cyp26a1* induction, demonstrating the requirement of this cis-regulatory module for full RA-responsive transcriptional activation. Single-cell transcriptomic profiling further shows that disruption of the enhancer-RARE module, or loss of *Cyp26a1* itself, alters RA-guided differentiation trajectories, reducing neuroectodermal progression while aberrantly expanding undifferentiated and mesenchymal-like populations. Together, our findings define an endogenous enhancer-RARE module that uniquely governs *Cyp26a1*-mediated RA clearance. By constraining RA availability during differentiation, this cis-regulatory mechanism functions as a crucial epigenetic checkpoint to stabilize developmental signaling and ensure the precise execution of proper cell fate trajectories.

RA, the active metabolite of vitamin A, is a pivotal morphogen that regulates embryonic patterning, regional identity, and lineage progression 1, 2, 3. In the nucleus, RA signaling is mediated by heterodimers of retinoic acid receptors (RARα, RARβ, and RARγ) and retinoid X receptors (RXRα, RXRβ, and RXRγ). Upon ligand activation, RAR/RXR complexes bind RA response elements (RAREs) and regulate transcriptional programs 4, 5. The biological effects of RA are highly sensitive to both dose and exposure duration (6). During pregnancy, vitamin A deficiency causes a spectrum of developmental defects collectively referred to as vitamin A deficiency syndrome 6, 7. Conversely, excessive exposure to RA or retinoids is teratogenic and can disrupt craniofacial, nervous system, and cardiovascular development, with some abnormalities overlapping those caused by vitamin A deficiency 6, 8. These observations underscore the importance of RA homeostasis, in which receptor-dependent transcription is coordinated with metabolic pathways that control ligand availability and thereby constrain the magnitude and duration of RA signaling. Defining how RA metabolism is integrated with the broader RA-responsive transcriptional program is therefore essential for explaining how this dose-sensitive signal directs reproducible early cell-fate transitions. Mouse embryonic stem cells, which provide a well-defined pluripotent state together with experimentally tractable differentiation and efficient genome engineering 9, 10, provide a suitable model for dissecting the relationship among RA signaling, metabolic feedback, and early fate progression 11, 12, 13, 14.

A key component of RA metabolic feedback is *Cyp26a1*, which encodes a cytochrome P450 enzyme that metabolizes RA into more polar derivatives and limits ligand availability (15). Previous studies have established that *Cyp26a1* is strongly inducible by RA and that RARE-containing regulatory sequences contribute to this response 16, 17. Distal cis-regulatory elements such as enhancers can support rapid and robust gene activation by recruiting stimulus-responsive transcription factors, acquiring active chromatin features, and establishing physical contacts with target promoters 18, 19, 20, 21. One hallmark of active enhancers is H3K27 acetylation (H3K27ac), which is frequently deposited by transcriptional coactivators such as the lysine acetyltransferase p300. In RA signaling, RAR/RXR complexes bind sequence-specific RAREs and can recruit coactivators that promote local chromatin activation 22, 23. However, the endogenous cis-regulatory architecture that couples RA receptor occupancy to *Cyp26a1* activation during pluripotent cell differentiation remains incompletely defined. It is also unclear whether specific RARE motifs within distal regulatory elements are required for full *Cyp26a1* induction and how disruption of this regulatory feedback affects the allocation of early differentiation states.

Here, we integrate time-resolved bulk and single-cell transcriptomics with cis-regulatory profiling and genetic perturbation to define an endogenous RA-responsive regulatory module at the *Cyp26a1* locus in mESCs. Temporal profiling identifies *Cyp26a1* as one of the most strongly induced genes during RA-driven differentiation. Among genes involved in RA synthesis and metabolism, *Cyp26a1* is distinguished by the formation of a highly RA-responsive distal enhancer. This enhancer contains core RARE motifs and is activated following RA exposure through RAR/RXR occupancy, p300 recruitment, H3K27ac accumulation, and increased enhancer-promoter interaction. Genetic disruption of the enhancer or its RARE motifs attenuates *Cyp26a1* induction and alters RA-responsive transcriptional programs. At single-cell resolution, perturbation of *Cyp26a1* or its enhancer-RARE module changes the distribution of early differentiation states, reducing neuroectodermal progression while increasing undifferentiated and mesenchymal-like populations. Together, these findings define an endogenous enhancer-RARE module that links ligand-responsive chromatin regulation to *Cyp26a1*-dependent RA metabolic feedback and contributes to appropriate cell fate progression during pluripotent cell differentiation.

## Results

### *Cyp26a1* acts as the central homeostatic effector in a phased RA-driven transcriptome remodeling

RA elicits time-dependent transcriptional responses that are critical for its developmental functions 24, 25. Defining the temporal dynamics of RA signaling in embryonic stem cells therefore provides a foundation for understanding how RA metabolism is coordinated with lineage-associated transcriptional networks. To characterize RA-driven transcriptional dynamics in mESCs, we established an RA time course, performed bulk RNA-seq at 0, 12, 24, 48, and 72 h, and generated single-cell transcriptomes at selected time points to analyze accompanying cell-state transitions (Fig. 1*A*). Time-resolved differential expression analysis revealed extensive transcriptional remodeling throughout differentiation. Each adjacent interval, including 0 to 12 h, 12 to 24 h, 24 to 48 h, and 48 to 72 h, showed extensive gene activation and repression (Fig. 1*B*), while comparisons with the 0 h state revealed progressively greater transcriptional divergence at later time points (Fig. S1*A*). These molecular changes were evidenced by RA-induced morphological reorganization and a progressive decline in alkaline phosphatase (AP) staining, consistent with exit from the undifferentiated state (Figs. 1*C* and S1*B*). Genes involved in RA synthesis, transport, binding, and metabolism also displayed marked temporal changes (Fig. S1*C*), consistent with coordinated remodeling of RA metabolic and homeostatic programs following RA exposure.

**Figure 1:**
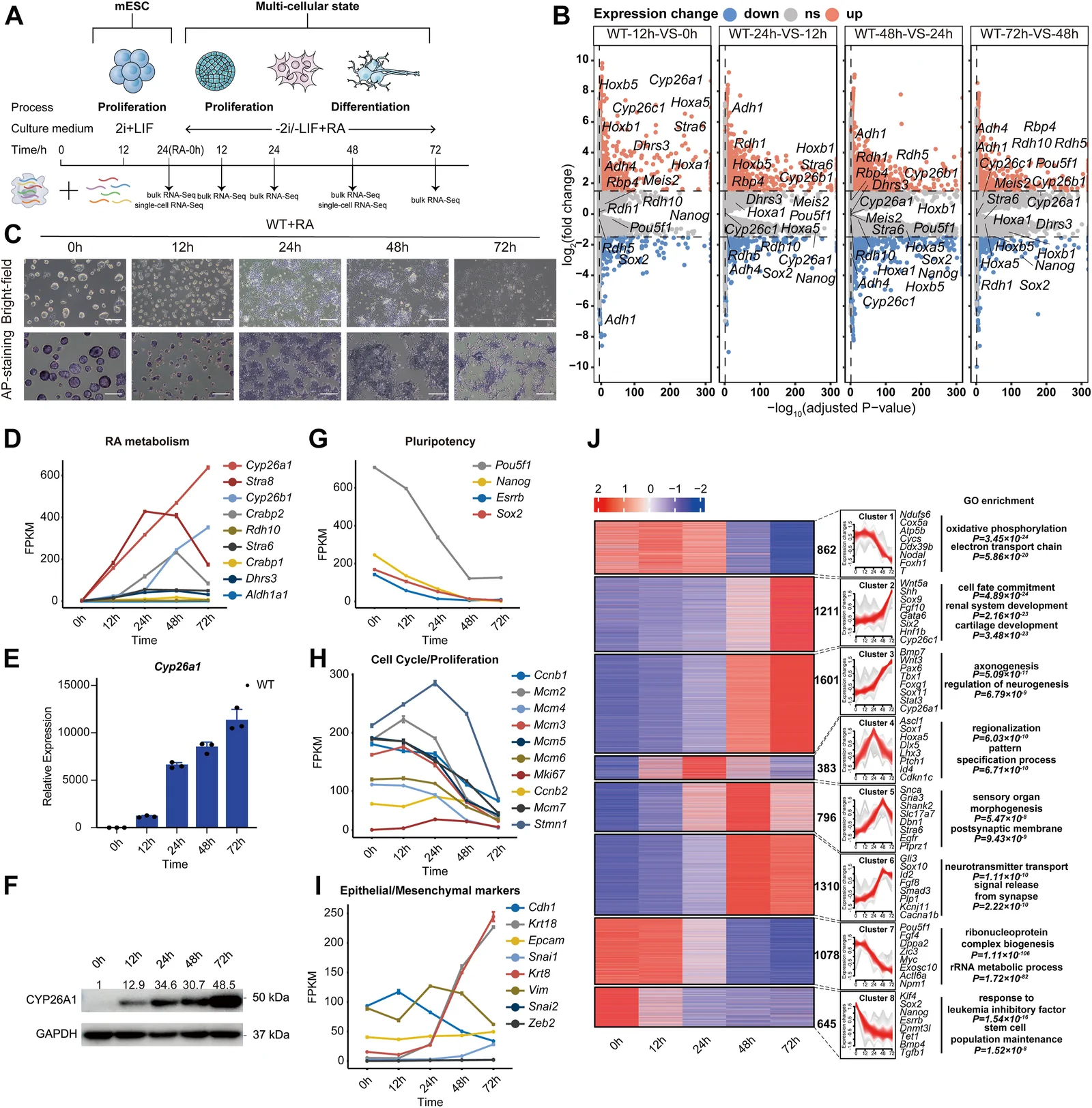
**RA triggers phased transcriptome remodeling and significantly activates*****Cyp26a1*****.***A*, schematic of the experimental workflow and sampling strategy. Wild-type mESCs were maintained under 2i + LIF conditions and then switched to -2i/-LIF medium supplemented with retinoic acid, defined as 0 h, to initiate differentiation. Bulk RNA-seq and single-cell RNA-seq were performed at the indicated time points. *B*, Volcano plots of differential gene expression between adjacent time points (12 h *versus* 0 h, 24 h *versus* 12 h, 48 h *versus* 24 h, and 72 h *versus* 48 h). The x-axis shows −log10(adjusted *p* value), and the y-axis shows log2(fold change). Significantly upregulated genes are shown in red, downregulated genes in blue, and genes not meeting the predefined DEG criteria are shown in grey. Selected genes are annotated. *C*, representative bright-field images (*top*) and alkaline phosphatase (AP) staining (*bottom*) of WT cells at the indicated time points after RA exposure. Scale bars, 200 μm. *D*, time-course expression profiles (FPKM) derived from bulk RNA-seq. Representative RA signaling transport/metabolism-associated genes. *E*, RT-qPCR analysis of *Cyp26a1* mRNA across the RA time course in WT mESCc. Data are shown as mean ± SD from three independent replicates. *F*, Western blot analysis of CYP26A1 protein abundance in WT mESCs. GAPDH was used as a loading control. Relative CYP26A1 levels are indicated above the blot. *G*, time-course expression profiles of representative pluripotency-associated genes derived from bulk RNA-seq. *H*, time-course expression profiles of representative cell cycle- and proliferation-associated genes, including *Ccnb1*, *Ccnb2*, *Mcm2-7*, *Mki67*, and *Stmn1. I*, time-course expression profiles of representative epithelial- and mesenchymal-associated genes, including *Cdh1*, *Krt8*, *Krt18*, *Epcam*, *Snai1*, *Snai2*, *Vim*, and *Zeb2. J*, unsupervised clustering of genes showing significant temporal regulation across the bulk RNA-seq time course. Z-score-normalized expression values are displayed as a heatmap and partitioned into eight modules. For each module, the average expression trajectory is shown in red together with individual gene trajectories in grey. Representative genes and selected GO BP terms are shown for each cluster.

Within this response, we found that *Cyp26a1* emerged as one of the most strongly induced genes and showed pronounced activation among the RA metabolism-associated genes, with its mRNA levels increasing markedly over time (Fig. 1*D*). This pattern was corroborated at both transcript and protein levels by RT-qPCR and Western blotting (Fig. 1, *E* and*F*). Concomitantly, expression of the core pluripotency regulators *Pou5f1*, *Nanog*, *Sox2*, and *Esrrb* progressively declined (Fig. 1*G*). Cell cycle- and proliferation-associated genes (including members of the Mcm family, *Mki67*, and cyclins) were extensively downregulated (Fig. 1*H*), consistent with reduced proliferative activity during exit from pluripotency. Epithelial- and mesenchymal-associated genes (*Epcam*, *Cdh1*, *Krt8/18*, *Vim*, and the *Snai/Zeb* family genes) underwent dynamic changes (Fig. 1*I*), further indicating coordinated remodeling of cell-state-associated transcriptional programs during RA-induced differentiation.

To delineate the RA-induced transcriptional program, we clustered time-series differentially expressed genes into eight modules with distinct temporal kinetics (Fig. 1*J*). These modules covered a broad functional spectrum. Clusters 1 and 7 were associated primarily with metabolic and biosynthetic functions, including oxidative phosphorylation and ribonucleoprotein complex biogenesis. Developmental processes and patterning-related signatures were enriched in Clusters 2 and 4, including cell fate commitment, pattern specification and regionalization. Neural-associated programs were concentrated in Clusters 3, 5 and 6, with enrichment for axonogenesis, regulation of neurogenesis, and synaptic signaling. In agreement with these temporal modules, Gene Ontology (GO) analysis showed that genes induced by RA were preferentially associated with neural development and patterning, whereas downregulated genes were enriched for immune-related, adhesion, and migration-associated processes (Fig. S1, *D* and *E*). HOX family genes exhibited RA-dependent induction with temporal progression (Fig. S1*F*), consistent with progressive engagement of regionalization and patterning features. Together, these data establish that RA induces robust, phased transcriptome remodeling in mESCs, characterized by activation of RA metabolic programs, strong induction of *Cyp26a1* and progressive engagement of patterning- and neural-associated transcriptional modules.

### RA guides early lineage commitment preferentially toward a neuroectoderm fate

RA-induced exit from pluripotency does not produce a uniform transitional state 1, 26. Instead, early differentiation rapidly generates divergent lineage trajectories, which differ in their responsiveness to extrinsic signals such as RA and in the retention of intrinsic pluripotency and self-renewal programs 26, 27, 28. To determine how RA remodels the early fate landscape in WT mESCs, we first examined differentiation-related transcriptional programs using the bulk RNA-seq time series. Heatmap analysis of lineage markers revealed rapid and temporally ordered activation of gene sets associated with pan-ectodermal, neuroectodermal, surface ectodermal, mesodermal, endodermal and mesenchyme-associated states following RA exposure (Fig. 2*A*), indicating broad activation of lineage-associated transcriptional programs. Consistently, signature enrichment analysis revealed a progressive decline in pluripotency markers, accompanied by increased ectodermal, endodermal, mesodermal and primitive endoderm signatures (Fig. S2*A*). Functional enrichment analysis further highlighted differentiation-related processes, including stem cell differentiation, epithelial–mesenchymal transition (EMT), anterior–posterior patterning, and regulation of neurogenesis (Fig. S2*B*). These transcriptional changes were accompanied by dynamic remodeling of developmental-state-associated gene programs. Pluripotency-associated genes declined over time, whereas epiblast-like, visceral endoderm and anterior visceral endoderm-like, and primitive streak-associated genes exhibited distinct transient or stage-specific induction patterns during RA treatment (Fig. S2*C*).

**Figure 2:**
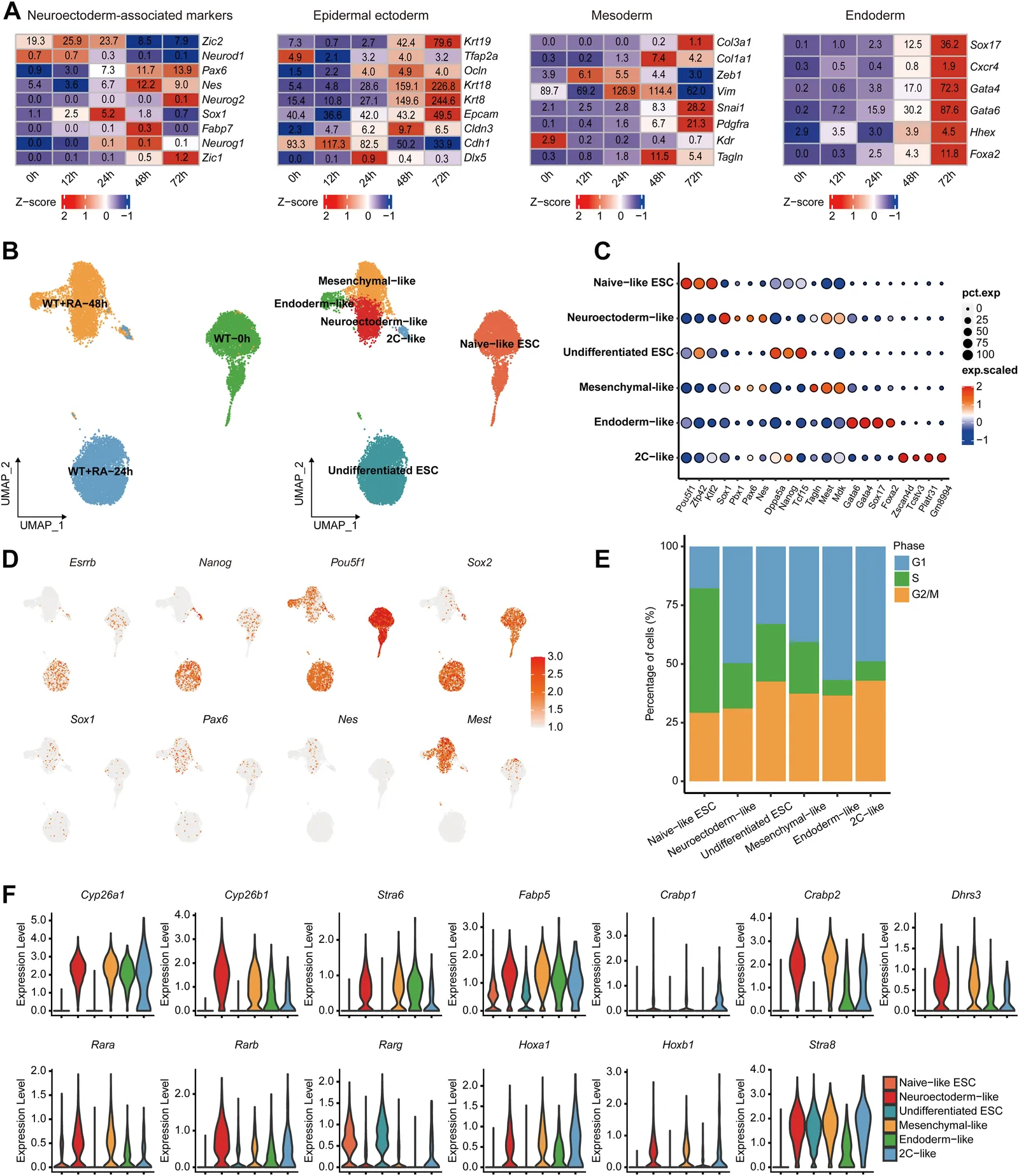
**RA induces ESCs toward a neuroectoderm-like differentiation trajectory.***A*, heatmaps showing temporal expression of selected developmental-state-associated marker genes during RA-induced differentiation. Representative genes associated with ectodermal, epidermal ectodermal, mesodermal, and endodermal programs are shown across 0, 12, 24, 48, and 72 h. Numerical values indicate expression levels, and colors represent Z-score-normalized relative expression. *B*, UMAP of single-cell RNA profiles (dots) of WT-0 h, ESC + RA-24 h and WT + RA-48 h. *Left*, cells colored by sample and time point. *Right*, cells are colored by annotated transcriptional states. Each dot represents one cell. *C*, dot plot summarizing marker gene expression across the annotated cell states. Color: average gene expression; size: proportion of cells expressing the gene. *D*, feature plots showing expression of representative pluripotency-associated genes and neuroectoderm-associated markers, together with Mest, across the integrated UMAP. *E*, cell-cycle phase composition across the annotated cell states, displayed as the fraction of cells assigned to G1, S or G2/M phase. *F*, Violin plots of RA uptake/transport and metabolism genes, retinoic acid receptors, and representative RA-responsive/axial markers across the annotated cell states.

We then characterized RA-induced fate diversification at single-cell resolution. Integrated UMAP analysis revealed clear separation of cell populations following RA treatment and identified multiple molecularly defined cell states. These included ESC-related populations (undifferentiated and naïve-like) and differentiated populations (neuroectoderm-like, endoderm-like, and mesenchymal-like), as well as a 2C-like population (Fig. 2*B*). These annotations were supported by the expression of stage- and lineage-associated marker genes (Fig. 2*C*). The neuroectoderm-like population expressed neural markers, including *Sox1*, *Pax6*, and *Nes*, and was clearly separated from ESC-associated populations that retained high expression of pluripotency factors such as *Esrrb*, *Nanog*, *Pou5f1*, and *Sox2* (Figs. 2*D* and S2*D*). These patterns indicate that RA-induced early differentiation primarily follows a neuroectoderm-like trajectory. Cell-cycle phase distributions also differed significantly among the identified populations, consistent with extensive remodeling of cellular states during differentiation (Fig. 2*E*).

To relate fate branching to RA responsiveness, we examined RA metabolism and signaling genes across the identified cell states. Genes involved in RA catabolism and metabolism, including *Cyp26a1*, *Cyp26b1*, and *Dhrs3*, RA uptake and intracellular binding factors, including *Stra6*, *Crabp1*, *Crabp2*, and *Fabp5*, and RA receptors, including *Rara*, *Rarb*, and *Rarg*, showed population-dependent expression patterns. Canonical RA-responsive genes, including *Hoxa1*, *Hoxb1* and *Stra8*, were likewise differentially distributed among cell populations (Fig. 2*F*). Together, these analyses establish a cell-state-resolved reference framework for evaluating how perturbations of RA metabolism alter the distribution of early differentiation states and reshape RA-responsive fate progression.

### *Cyp26a1* deletion disrupts RA-responsive transcription and redirects cell fate trajectories

To investigate how *Cyp26a1*-dependent RA metabolism influences RA-responsive differentiation, we performed time-resolved transcriptomic and phenotypic comparisons between wild-type (WT) and *Cyp26a1* knockout (*Cyp26a1*-KO) mESCs during RA treatment. Bulk RNA-seq confirmed strong induction of *Cyp26a1* in WT cells, whereas *Cyp26a1* expression remained undetectable in KO cells throughout the 72-h time course (Fig. 3*A*). Consistent with this result, CYP26A1 protein was readily detected in WT cells after 48 h of RA treatment but remained absent in *Cyp26a1*-KO cells (Fig. 3*B*). Comparisons between KO and WT cells at 0, 12, 24, 48 and 72 h revealed extensive differential gene expression across the time course, indicating sustained transcriptional consequences of *Cyp26a1* loss during RA-induced differentiation (Figs. 3*C* and S3*A*). Analysis of core pluripotency regulators further supported altered differentiation kinetics. Although *Pou5f1* and *Nanog* declined during RA treatment in both genotypes, their temporal expression patterns differed between KO and WT cells (Fig. 3*D*). Similar differences were observed across a broader pluripotency-associated gene set, including *Klf4*, *Esrrb*, *Sox2*, and *Zfp42* (Fig. S3*B*). Consistently, *Cyp26a1*-KO cells exhibited stronger AP staining signals and displayed distinct morphology after 48 h of RA treatment. Quantification showed comparable AP staining at baseline but significantly higher AP signals in KO cells at 48 h, supporting delayed or incomplete exit from the undifferentiated state (Fig. 3*E*).

**Figure 3:**
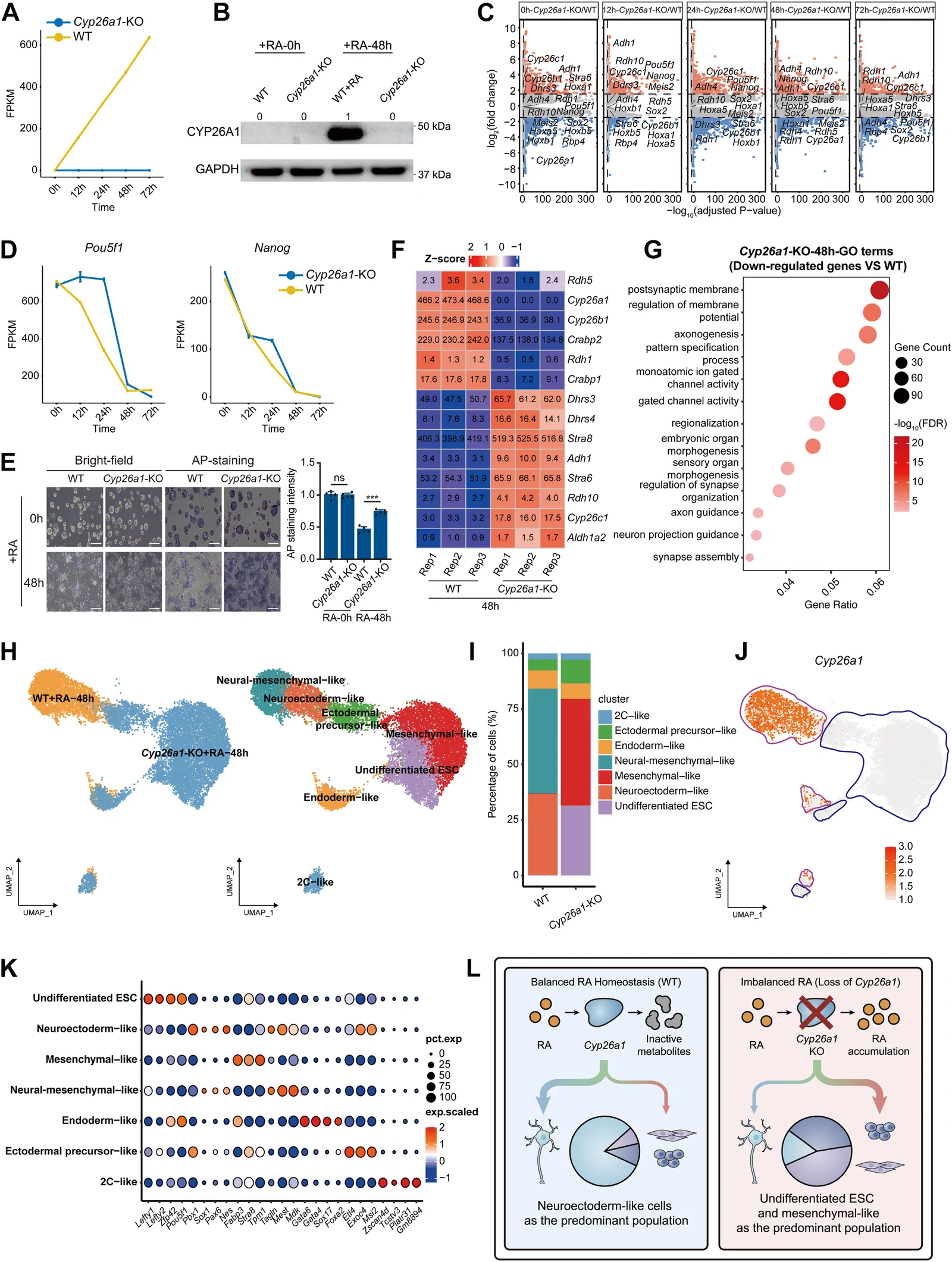
***Cyp26a1* knockout reshapes RA-induced transcriptomes and alters early ESC lineage fates.***A*, bulk RNA-seq time-course showing *Cyp26a1* expression (FPKM) in WT and *Cyp26a1*-KO cells at the indicated time points after RA exposure (0, 12, 24, 48 and 72 h). *B*, Western blot showing CYP26A1 protein in WT and *Cyp26a1*-KO cells at 0 h and 48 h after RA treatment. GAPDH serves as a loading control. Relative CYP26A1 protein levels are indicated above the blot. *C*, volcano plots of differential gene expression comparing *Cyp26a1*-KO to WT at each time point (0, 12, 24, 48 and 72 h). The x-axis indicates −log10(adjusted *p* value) and the y-axis indicates log2(fold change). *D*, bulk RNA-seq time-course expression profiles (FPKM) of representative pluripotency-associated genes in WT and *Cyp26a1*-KO cells across the RA time course. *E*, representative bright-field images (*left*) and AP staining (*middle*) of WT and *Cyp26a1*-KO cultures at 0 h and 48 h after RA treatment. *Right*, quantification of AP staining intensity. Data are shown as mean ± SD; ns, not significant; ∗∗∗*p* < 0.001. Scale bars, 150 μm. *F*, heatmap of RA uptake/transport and metabolism-associated transcripts at 48 h after RA exposure in WT and *Cyp26a1*-KO cells (three replicates each, Rep1-Rep3). Values are displayed as row-wise Z-scores. *G*, GO-BP enrichment analysis of genes downregulated in *Cyp26a1*-KO relative to WT at 48 h. Dot size indicates the number of genes (Gene Count), and dot color indicates −log10(FDR). *H*, UMAP embedding of single-cell RNA-seq profiles collected at 48 h after RA treatment from WT and *Cyp26a1*-KO cells. *Left*, cells are colored by genotype/condition (WT + RA-48 h, *yellow*; *Cyp26a1*-KO + RA-48 h, *blue*). *Right*, the same embedding is colored by annotated cell states. Each dot represents one cell. *I*, cell-state composition of WT and *Cyp26a1*-KO samples at 48 h after RA treatment, shown as the percentage of cells assigned to each annotated cell state. *J*, feature plot showing per-cell *Cyp26a1* expression projected onto the UMAP in (*H*). Color intensity indicates expression level. *K*, dot plot summarizing marker gene expression across the annotated cell states. Dot color indicates scaled average expression, and dot size indicates the proportion of cells expressing each gene. *L*, schematic summary comparing RA homeostasis and the distribution of differentiation states in WT and *Cyp26a1*-KO cells.

Given the role of *Cyp26a1* in RA clearance, we examined genes involved in RA metabolism, transport, and intracellular binding. In addition to loss of *Cyp26a1*, *Cyp26a1*-KO cells showed coordinated changes in multiple RA-handling genes across the time course (Figs. 3*F* and S3, *C, D*). Downstream RA-responsive genes and HOX family genes also displayed genotype-dependent temporal differences (Fig. S3, *E* and *F*), consistent with the corresponding single-cell expression patterns of representative HOX genes (Fig. S3*G*). At the functional level, genes downregulated in *Cyp26a1*-KO cells were significantly enriched for neural-associated and patterning-related processes, including axonogenesis, pattern specification, regionalization, and synaptic organization (Fig. 3*G*). Similar attenuation of neural-associated and patterning programs was observed at 24 and 72 h (Fig. S4, *A* and *B*). By contrast, genes upregulated in *Cyp26a1*-KO cells were enriched for distinct functional categories at different time points (Fig. S4, *C–E*). These results indicate that loss of *Cyp26a1* reshapes RA-induced transcriptional programs, with a prominent reduction in gene programs associated with neural development and embryonic patterning.

To determine whether these transcriptional changes were reflected in cell-state composition, we performed single-cell RNA-seq of WT and *Cyp26a1*-KO cells after 48 h of RA treatment. The combined UMAP projection showed clear genotype-associated separation and resolved multiple transcriptionally defined populations (Fig. 3*H*). These populations included undifferentiated ESCs, neuroectoderm-like cells, neural-mesenchymal-like cells (co-expressing neural- and mesenchymal-associated markers, defined by *Sox1*, *Pax6*, *Nes*, *Tagln*, and *Mest*), mesenchymal-like cells, endoderm-like cells, ectodermal precursor-like cells (defined by *Etl4*, *Exoc4*, and *Pbx1*), and 2C-like cells. Cell-composition analysis showed a reduced neuroectoderm-like fraction together with expansion of mesenchymal-like and undifferentiated ESC populations in *Cyp26a1*-KO cells (Fig. 3*I*). Feature plots confirmed *Cyp26a1* expression in WT cells and its absence in KO cells at single-cell resolution (Fig. 3*J*). Population annotation was supported by marker expression profiles (Figs. 3*K* and S5, *A–H*), and lineage-associated signature scores differed between WT and *Cyp26a1*-KO cells (Fig. S5*I*). Together, these data show that loss of *Cyp26a1* alters RA-responsive transcriptional programs and shifts the distribution of early differentiation states during mESC differentiation (Fig. 3*L*).

### A distal *Cyp26a1* enhancer serves as an RA nuclear receptor-governed regulatory hub

The rapid cellular response to RA is reflected not only in transcriptional changes but also in dynamic remodeling of regulatory chromatin 29, 30. Ligand-activated nuclear receptors bind sequence-specific regulatory elements, recruit transcriptional coactivators, and promote active enhancer features such as H3K27ac, often in association with enhancer-promoter communication within three-dimensional chromatin organization 21, 31. We therefore asked whether RA-responsive chromatin remodeling occurs at genes involved in RA synthesis and metabolism. To address this, we systematically compared H3K27ac profiles across representative RA metabolism-associated loci in untreated and RA-treated WT mESCs, together with RARα and RXRα occupancy. Among the loci examined, *Cyp26a1* showed the most prominent RA-responsive chromatin change. A distal region at the *Cyp26a1* locus acquired strong H3K27ac enrichment following RA exposure and was co-occupied by RARα and RXRα, leading us to prioritize this region as a candidate RA-responsive enhancer (Fig. 4*A*).

**Figure 4:**
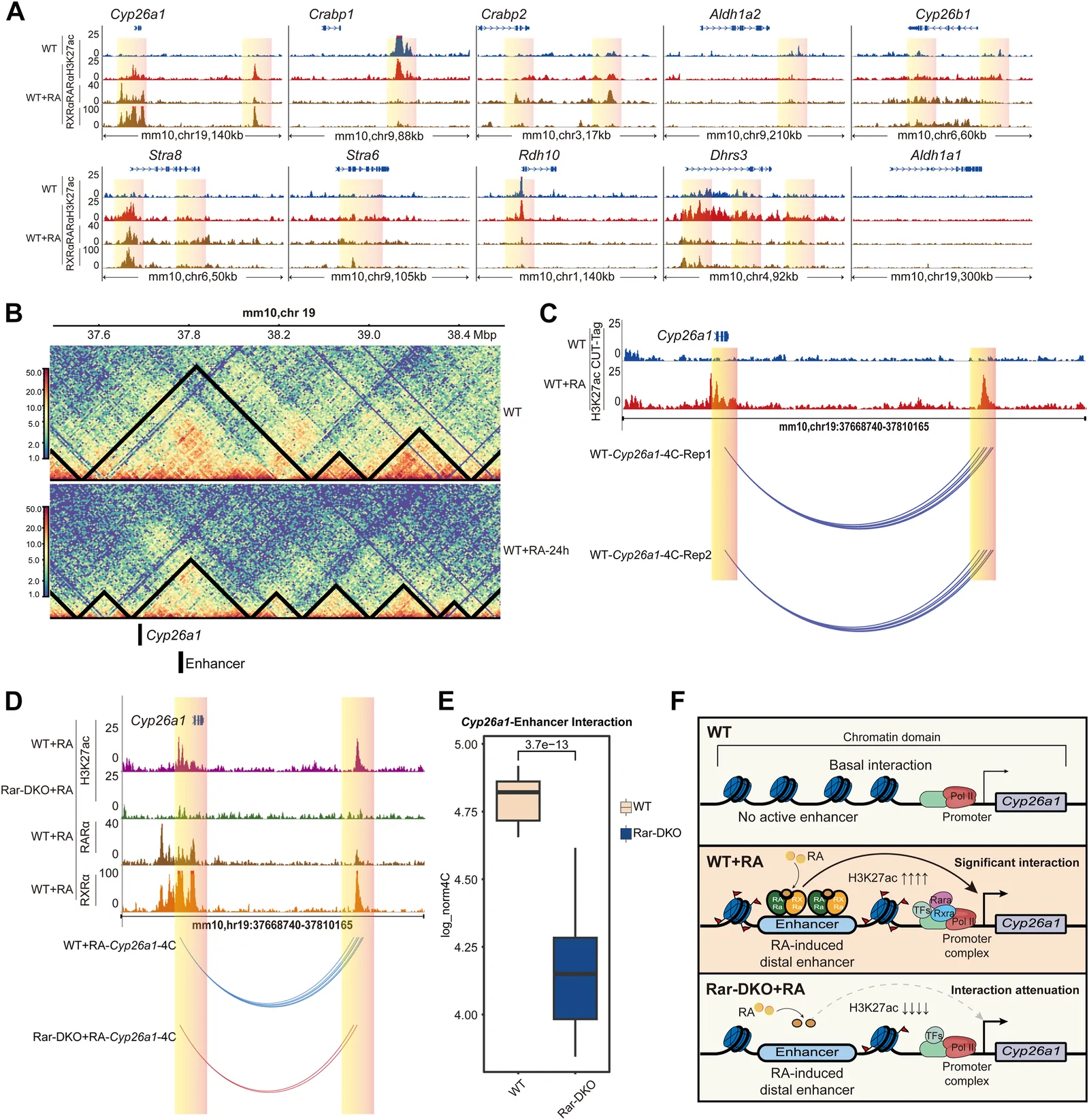
**The RA nuclear receptor promotes significant interaction between a distal-specific enhancer and the *Cyp26a1* promoter.***A*, genome browser views showing H3K27ac ChIP–seq signal in WT and WT + RA (24 h), together with RARα and RXRα ChIP-seq signal in WT + RA (24 h), across the indicated RA uptake/transport and metabolism-associated genes. Shaded vertical bands highlight the selected regulatory regions. *B*, Hi-C contact maps across the Chr19 region encompassing *Cyp26a1* in WT and WT + RA (24 h). Color intensity indicates contact frequency (scale shown). Black triangle outlines indicate topologically associating domains (TADs) as drawn on the maps. The positions of *Cyp26a1* and the distal enhancer are indicated below the maps. *C*, *Cyp26a1* locus tracks showing H3K27ac ChIP-seq in WT and WT + RA (24 h, top) and 4C interaction profiles generated using the *Cyp26a1* promoter as the viewpoint (bottom; two biological replicates). Shaded vertical bands mark the viewpoint-proximal region and the distal interacting region. *D*, *Cyp26a1* locus tracks showing H3K27ac ChIP-seq in WT + RA and Rar-DKO + RA, together with RARα and RXRα ChIP-seq in WT + RA (*top*). *Bottom*, 4C interaction profiles generated using the *Cyp26a1* promoter as the viewpoint in WT + RA and Rar-DKO + RA. Shaded vertical bands denote the interacting regions used for visualization. Cells were analyzed after 24 h of RA treatment. *E*, Box plots summarizing the *Cyp26a1* promoter-enhancer 4C interaction signal in WT and Rar-DKO under RA treatment. Centre line, median; box, interquartile range; whiskers, range. *F*, schematic summary of distal enhancer activation, H3K27ac enrichment, and enhancer-promoter interaction in WT, WT + RA, and Rar-DKO + RA conditions.

We next examined the local chromatin environment of this element. Hi-C contact maps on chr19 showed that the *Cyp26a1* promoter and distal enhancer reside within the same topologically associating domain (TAD), providing a chromatin context compatible with intra-domain regulation (Fig. 4*B*). We then performed 4C-seq using the *Cyp26a1* promoter as the viewpoint. Replicate 4C profiles identified reproducible contacts between the *Cyp26a1* promoter and the distal region containing the RA-induced H3K27ac peak (Fig. 4*C*), supporting a functionally relevant promoter–enhancer connection at this locus.

To test whether activation of this distal element and its interaction with the *Cyp26a1* promoter depend on RA receptor activity, we analyzed *Rara*/*Rxra* double-knockout (Rar-DKO) cells following RA treatment. Compared with WT cells, Rar-DKO cells showed a marked reduction in H3K27ac enrichment at the distal *Cyp26a1* enhancer (Fig. 4*D*). In parallel, the interaction between this enhancer and the *Cyp26a1* promoter was substantially weakened, as confirmed by quantitative analysis (Fig. 4*E*). Together, these data demonstrate that RARα/RXRα activity is required for full activation of the distal *Cyp26a1* enhancer and for efficient enhancer–promoter communication in response to RA (Fig. 4*F*).

### RAREs within the enhancer license RA-driven *Cyp26a1* induction *via* p300-catalyzed H3K27ac

H3K27ac is a hallmark of active enhancers (32), and its deposition is mediated in part by histone acetyltransferases (HATs) 33, 34. Ligand-activated RAR/RXR complexes bind retinoic acid response elements (RAREs) and recruit transcriptional coactivators, including histone acetyltransferase-containing complexes, to promote local histone acetylation and transcriptional activation 5, 35. To identify functional RAREs within the distal enhancer, we performed motif scanning using multiple RAR/RXR consensus models. Three high-scoring motif predictions were initially obtained. However, the first and third motif models mapped to the same genomic sequence, representing redundant predictions of a single site, which we designated RARE1. The second motif model identified a distinct sequence, designated RARE2. Thus, two nonredundant candidate RAREs were defined within the enhancer (Fig. 5, *A* and *B*). Because RA binding induces conformational changes in RAR/RXR that favor corepressor release and coactivator recruitment, including SRC/p160 family members and CBP/p300, we next assessed whether RAR/RXR is associated with p300-containing complexes 36, 37. In RA-treated WT cells, p300 co-immunoprecipitated with RARα and RXRα, and reciprocal p300 immunoprecipitation recovered both receptors. These interactions were absent in Rar-DKO cells and were not detected in IgG controls, supporting the presence of RARα, RXRα, and p300 within the same protein complex following RA stimulation (Fig. 5*C*).

**Figure 5:**
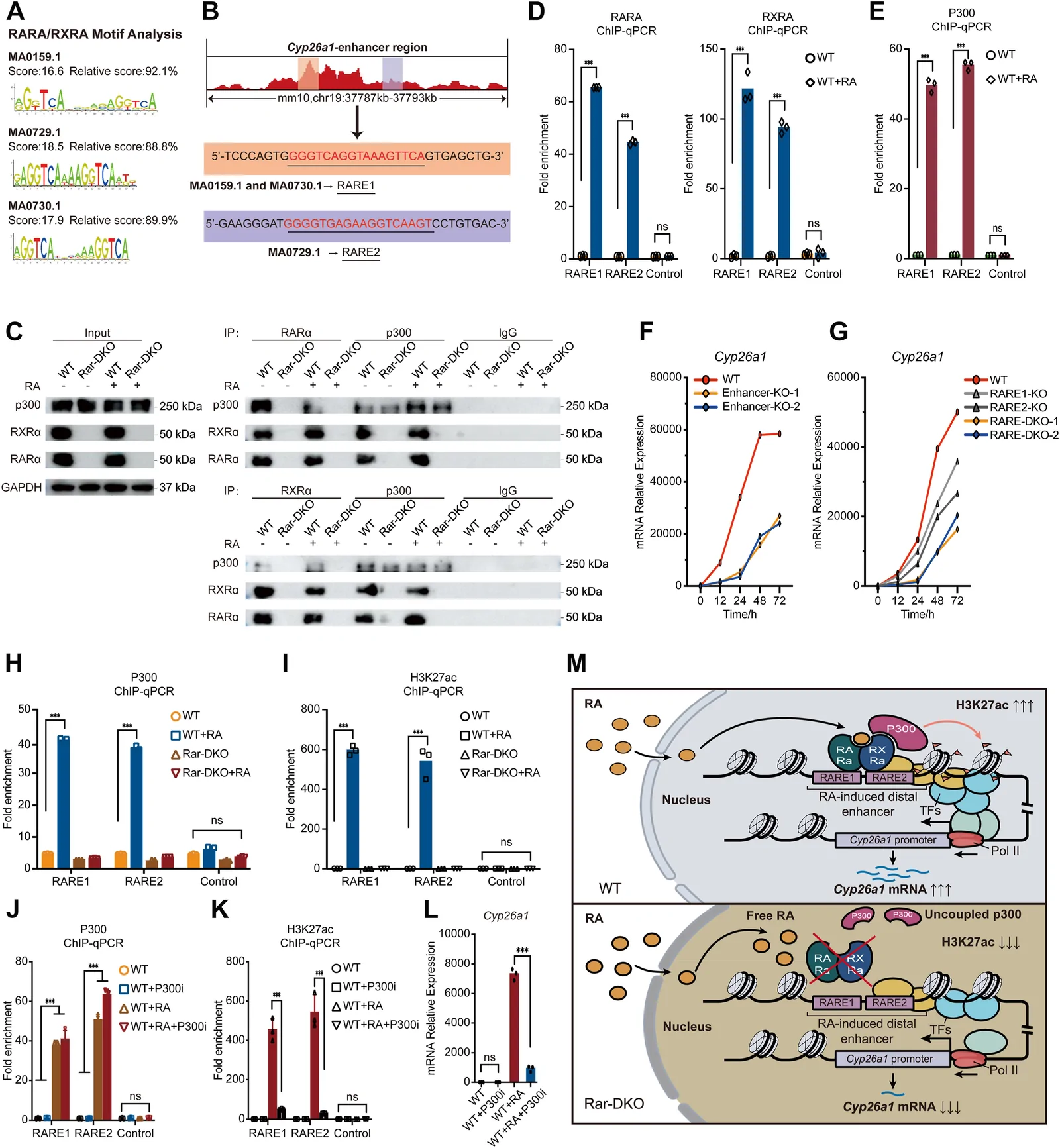
**RARα/RXRα promotes activation of the *Cyp26a1* distal enhancer through p300 recruitment.***A*, in silico identification of candidate RAR/RXR-binding motifs within the *Cyp26a1* distal enhancer. Representative RARE motif sequence logos are shown. *B*, genome browser view of the *Cyp26a1* distal enhancer (track shown in *red*) with the positions of two retinoic acid response elements (RARE1 and RARE2) highlighted (shaded boxes). The corresponding sequences are shown below. *C*, co-immunoprecipitation analysis of RARα, RXRα and p300 in WT and Rar-DKO cells under the indicated conditions. Rar-DKO cells and IgG immunoprecipitation were included as negative controls. Molecular weight markers are indicated. *D*, ChIP-qPCR quantification of RARα (*left*) and RXRα (*right*) occupancy at RARE1, RARE2, and a negative-control region (*Control*) in WT cells ± RA (48 h). Bars indicate the mean and symbols indicate individual biological replicates (n = 3). Statistical comparisons are shown by brackets (two-sided unpaired t tests); ∗∗∗*p* < 0.001; ns, not significant. The negative-control region was located at mm10, chr19:37,944,163-37,944,295. *E*, ChIP-qPCR quantification of P300 enrichment at RARE1, RARE2, and Control in WT cells ± RA (48 h). ∗∗∗*p* < 0.001; ns, not significant. Cells were analyzed after 48 h of RA treatment. *F*, RT-qPCR quantification of *Cyp26a1* mRNA across the RA time course in WT and two independent enhancer knockout clones (Enhancer-KO-1 and Enhancer-KO-2) mESCc. Data are shown as mean ± SD, n = 3 independent replicates; two-sided unpaired t tests. *G*, RT-qPCR quantification of *Cyp26a1* mRNA across the RA time course in WT, individual RARE knockout lines (RARE1-KO and RARE2-KO) and two independent double-RARE knockout clones (RARE-DKO-1 and RARE-DKO-2) mESCs. *H*, ChIP-qPCR quantification of p300 enrichment at RARE1, RARE2 and Control in WT and Rar-DKO cells ± RA (groups as indicated). Bars indicate the mean and symbols indicate individual biological replicates (n = 3). Statistical comparisons are shown by brackets (two-sided unpaired t tests); ∗∗∗*p* < 0.001; ns, not significant. Cells were analyzed after 48 h of RA treatment. *I*, ChIP-qPCR quantification of H3K27ac enrichment at RARE1, RARE2 and Control in WT and Rar-DKO cells ± RA. ∗∗∗*p* < 0.001; ns, not significant. Cells were analyzed after 48 h of RA treatment. *J*, ChIP-qPCR quantification of p300 enrichment at RARE1, RARE2, and Control in WT cells treated with RA in the presence or absence of the p300 catalytic inhibitor A-485 (P300i), as indicated. *K*, ChIP-qPCR quantification of H3K27ac enrichment at RARE1, RARE2, and Control under the same treatment conditions as in (*J*). *L*, RT-qPCR quantification of *Cyp26a1* mRNA expression in WT cells treated with RA and/or A-485 (P300i), as indicated. *M*, schematic model summarizing RAR/RXR-dependent p300 recruitment, H3K27ac enrichment, and transcriptional activation of *Cyp26a1* at the RA-responsive distal enhancer. TFs, transcription factors; Pol II, RNA polymerase II.

We next tested receptor occupancy and p300 recruitment at RARE1 and RARE2 within the endogenous enhancer. ChIP-qPCR after RA treatment revealed specific enrichment of both RARα and RXRα at RARE1 and RARE2, whereas no enrichment was detected at the negative control locus (Fig. 5*D*). Similarly, p300 was also significantly enriched at the same sites (Fig. 5*E*), linking these enhancer-embedded motifs to receptor occupancy and coactivator recruitment. To evaluate functional requirement, we genetically perturbed the enhancer and quantified *Cyp26a1* induction over time. RT-qPCR showed that deletion of the distal enhancer (Enhancer-KO, mm10, chr19: 37,788,057–37,792,531) considerably attenuated RA-induced *Cyp26a1* expression (Fig. 5*F*). Deletion of either RARE1 or RARE2 reduced the magnitude of Cyp26a1 induction, whereas combined deletion of both motifs (RARE-DKO) produced a stronger reduction (Fig. 5*G*), indicating that both RAREs are required for full enhancer-dependent activation.

To determine whether p300 recruitment and enhancer acetylation depend on RARα/RXRα activity, we measured p300 occupancy and H3K27ac enrichment in Rar-DKO cells. RA treatment induced strong p300 recruitment to RARE1 and RARE2 in WT cells, whereas this recruitment was markedly reduced in Rar-DKO cells (Fig. 5*H*). H3K27ac enrichment at both RARE-containing regions was similarly diminished following *Rara*/*Rxra* deletion, while the negative control region remained largely unchanged (Fig. 5*I*). These findings place RARα/RXRα upstream of p300 recruitment and local enhancer acetylation.

To directly test whether p300 acetyltransferase activity is required for enhancer acetylation and *Cyp26a1* induction, we treated WT cells with the catalytic p300 inhibitor A-485 during RA stimulation. A-485 did not prevent p300 occupancy at RARE1 and RARE2, with robust p300 enrichment retained at both sites following RA treatment (Fig. 5*J*). In contrast, inhibition of p300 catalytic activity markedly reduced H3K27ac enrichment at both RAREs (Fig. 5*K*) and strongly suppressed RA-induced *Cyp26a1* expression (Fig. 5*L*). Together, these results support a model in which RA-bound RARα/RXRα complexes occupy RARE1 and RARE2, recruit p300, and promote local H3K27 acetylation to achieve full transcriptional activation of *Cyp26a1* (Fig. 5*M*).

### Deletion of the distal enhancer triggers global reprogramming of the RA-driven transcriptome and reshapes the early fate landscape

The results above establish *Cyp26a1* as an important regulator of RA homeostasis and early differentiation-state distribution in mESCs and identify a distal *cis*-regulatory module at the *Cyp26a1* locus that is activated in an RAR/RXR-dependent manner. We next asked whether this enhancer is required to maintain RA-induced *Cyp26a1* expression and downstream differentiation programs. To address this, we generated two independent mESC clones (Enhancer-KO) and compared them with WT cells during RA treatment. Time-series transcriptome analysis showed that enhancer deletion substantially reduced the magnitude of *Cyp26a1* induction relative to WT cells (Fig. 6*A*). Consistently, quantitative immunoblotting showed that CYP26A1 protein abundance was significantly reduced in Enhancer-KO cells after 48 h of RA treatment (Fig. 6*B*). Globally, Enhancer-KO cells displayed widespread differential expression relative to WT cells during RA treatment, with transcriptional differences becoming more pronounced over time (Fig. S6, *A* and *B*). Genes involved in RA metabolism and homeostasis also showed coordinated temporal changes in Enhancer-KO cells, with some variation among clones and time points (Figs. 6*C* and S6*C*).

**Figure 6:**
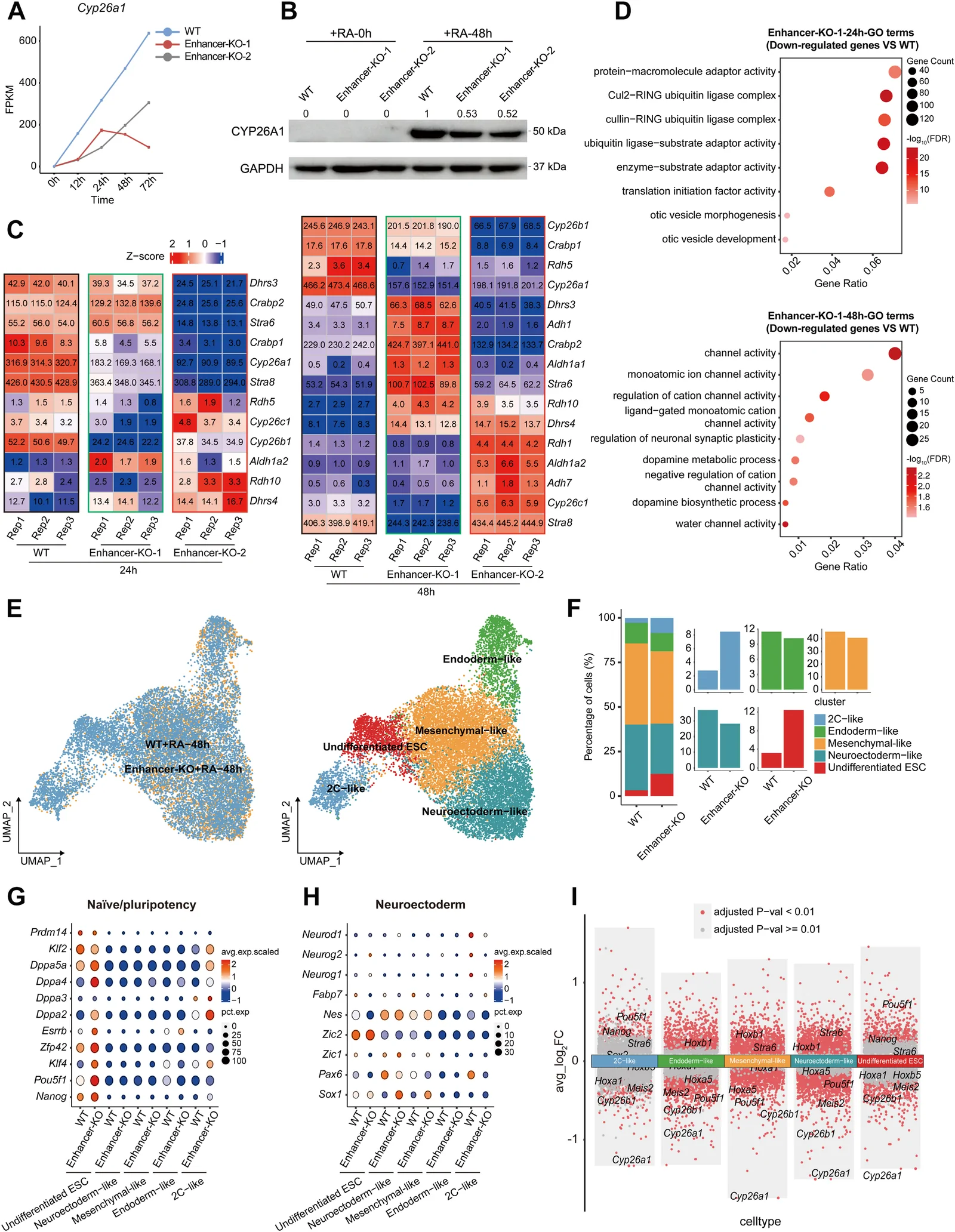
**Enhancer deletion significantly reduces RA-induced *Cyp26a1* transcription levels and disrupts ESC fate lineages.***A*, Bulk RNA-seq time course of *Cyp26a1* expression (FPKM) in WT and two independent distal-enhancer knockout clones (Enhancer-KO-1 and Enhancer-KO-2) at the indicated time points after RA exposure (0, 12, 24, 48 and 72 h). *B*, Western blot showing CYP26A1 in WT, Enhancer-KO-1 and Enhancer-KO-2 at 0 h and 48 h after RA exposure. GAPDH serves as a loading control; molecular weight markers are indicated. Relative CYP26A1 protein levels are indicated above the blot. *C*, heatmaps summarizing RA uptake/transport and metabolism-related transcripts at 24 h (*left*) and 48 h (*right*) after RA exposure in WT, Enhancer-KO-1 and Enhancer-KO-2 (n = 3, Rep1-Rep3). Numbers in tiles indicate FPKM values; colors indicate row-wise Z-score-normalized expression. *D*, GO-BP enrichment for genes downregulated in Enhancer-KO-1 relative to WT at 24 h (*top*) and 48 h (*bottom*) after RA exposure. Dot size indicates Gene Count, and dot color indicates −log10(FDR). *E*, UMAP embedding of single-cell RNA-seq profiles collected at 48 h after RA exposure from WT and enhancer knockout cells. *Left*, cells colored by genotype (WT + RA-48 h, *orange*; Enhancer-KO + RA-48 h, *blue*). *Right*, the same embedding colored by annotated cell states. *F*, cell-state composition at 48 h after RA exposure, shown as the percentage of cells assigned to each annotated state in WT and Enhancer-KO. Inset bar plots show selected cell-state proportions on expanded scales. *G*-*H*: Dot plots summarizing marker gene expression at 48 h after RA exposure across annotated cell states, separated by genotype (WT *versus* Enhancer-KO within each state). *G*, naïve pluripotency-associated markers. *H*, neuroectoderm-associated markers. *I*, Cell-state-stratified differential expression summary at 48 h after RA exposure. Points represent genes tested within each annotated cell state; *red* indicates adjusted *p* < 0.01 and grey indicates adjusted *p* ≥ 0.01. The y-axis shows the average log2 fold change between Enhancer-KO and WT cells.

GO enrichment analysis indicated that enhancer deletion altered RA-induced transcriptional programs in a time-dependent manner. At 48 h, genes downregulated in Enhancer-KO cells were prominently enriched for neural-related functional categories, including ion channel activity, neurotransmitter metabolism, and regulation of synaptic plasticity (Fig. 6*D*). Analyses of additional time points and both Enhancer-KO clones further identified recurrent enrichment of neurogenesis-, axonogenesis-, and ion channel-associated processes among downregulated genes (Fig. S7, *A* and *B*). In contrast, genes upregulated in Enhancer-KO cells were associated with RA response, retinoid metabolism, and related developmental or morphogenetic processes at selected time points (Fig. S7, *C* and *D*). Consistent with these functional shifts, time-course expression analyses showed altered kinetics of several patterning genes, including *Cdx2*, *Meis2* and multiple *Hox* genes, in Enhancer-KO cells compared with WT cells (Fig. S8, *A*–*E*). Developmental-state-associated signatures, including ectodermal, pluripotency, trophectodermal, endodermal, and mesodermal programs, also differed between genotypes over time (Fig. S8, *F* and *G*), indicating that enhancer deletion reshapes the transcriptional progression induced by RA.

We next examined whether these transcriptional alterations were reflected in early cell-state composition. Single-cell RNA-seq after 48 h of RA treatment, followed by joint UMAP analysis, resolved multiple distinct transcriptionally defined cell states, including undifferentiated ESCs, neuroectoderm-like cells, mesenchymal-like cells, endoderm-like cells, and 2C-like cells (Figs. 6*E* and S9*A*). Cell-composition analysis revealed clear genotype-dependent differences across these states. Compared with WT cells, Enhancer-KO cells showed increased proportions of undifferentiated ESC and 2C-like cells together with a reduced neuroectoderm-like population (Fig. 6*F*). Marker expression profiles supported these cell-state annotations (Figs. 6, *G*, *H* and S9, *B*, *C*). State-resolved differential expression analysis further showed that *Cyp26a1*, together with multiple genes associated with RA responsiveness and pattern formation, was altered in a cell-state-dependent manner (Figs. 6*I* and S9*D*). Further analysis demonstrated differential expression of genes involved in RA metabolism, receptor-mediated signaling, and downstream RA responses across distinct cellular states (Fig. S9*E*). Collectively, these data demonstrate that deletion of the distal *Cyp26a1* enhancer attenuates RA-induced *Cyp26a1* transcription and alters RA-related transcriptional programs, patterning gene dynamics and early fate-state composition during mESC differentiation.

### RAREs act as central genomic determinants that govern both RA-metabolic rewiring and fate redistribution

Given that the distal enhancer of *Cyp26a1* contains two functional RAREs, we next asked whether these motifs form the core *cis*-elements mediating the RA-dependent enhancer activity. To test this, we compared WT and RARE-DKO cells using time-series bulk transcriptomics and single-cell analysis. Bulk RNA-seq showed that RA-induced *Cyp26a1* expression was markedly attenuated in RARE-DKO cells relative to WT cells throughout the differentiation time course (Fig. 7*A*). Protein analysis was consistent with this result. After 48 h of RA treatment, robust CYP26A1 protein was detected in WT cells and cells lacking either RARE1 or RARE2 individually, whereas its abundance was substantially reduced in both RARE-DKO clones (Fig. 7*B*). These findings indicate that the two enhancer-embedded RAREs cooperate to support full RA-induced *Cyp26a1* activation. RA-responsive patterning genes *Hoxa1* and *Hoxb1* were rapidly induced in WT cells, whereas their induction was attenuated in RARE-DKO cells (Fig. 7*C*). Several additional *Hox* family members and RA-responsive genes, including *Cdx1*, *Meis2*, and *Stra8*, likewise displayed altered induction kinetics in the RARE-DKO background (Fig. S10, *A*–*E*). Corresponding to these changes, lineage-signature analysis indicated that RARE-DKO cells retained higher pluripotency features, whereas ectoderm-related scores were generally reduced and mesoderm-related and several other lineage-related scores were relatively increased, consistent with a shifted RA-driven differentiation trajectory (Fig. S10, *F*–*J*).

**Figure 7:**
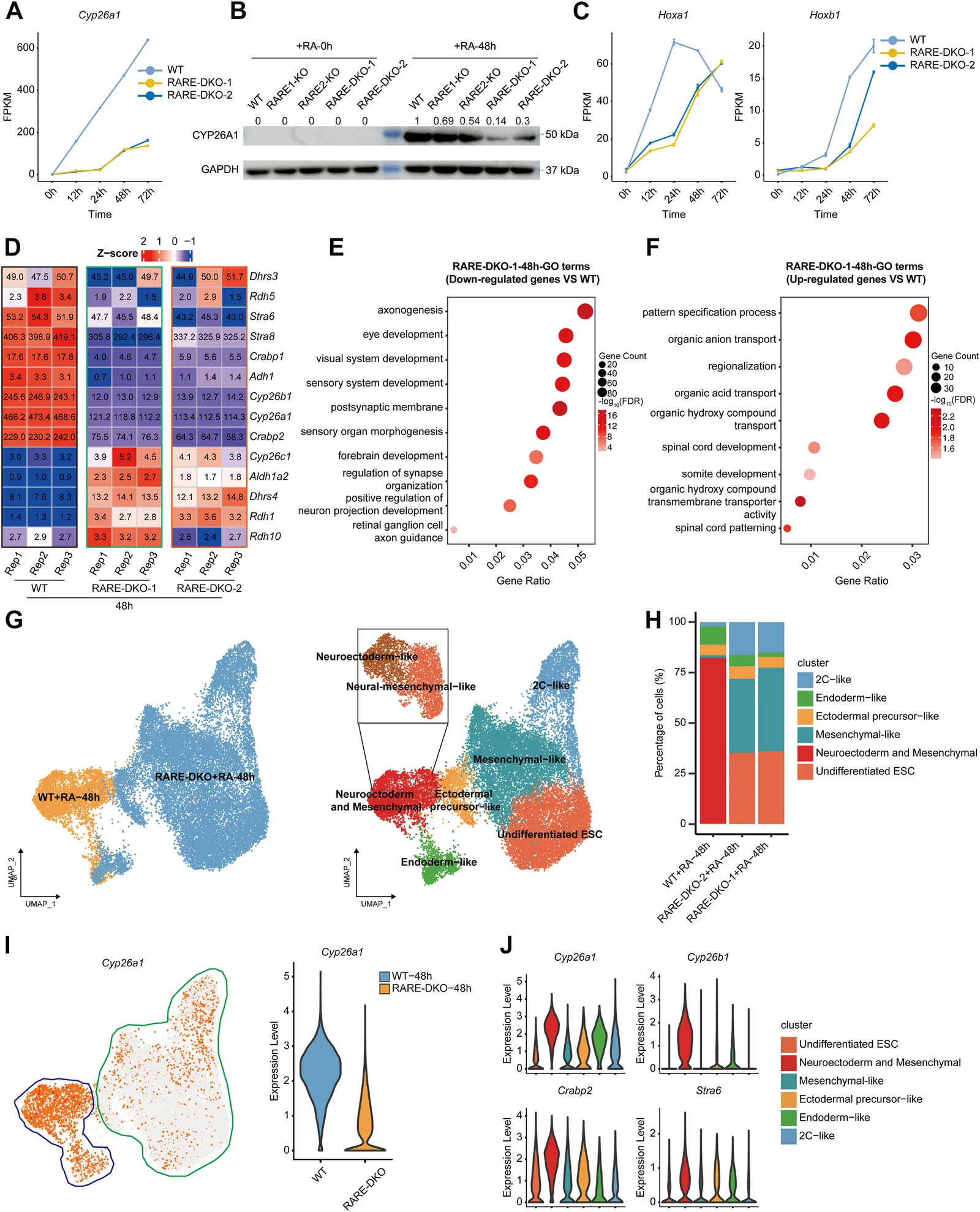
**Deletion of the enhancer-embedded RAREs attenuates RA-induced *Cyp26a1* expression and alters early differentiation states.***A*, bulk RNA-seq time course of *Cyp26a1* expression (FPKM) in WT and two independent RARE double-knockout lines (RARE-DKO-1 and RARE-DKO-2) at the indicated time points after RA exposure (0, 12, 24, 48 and 72 h). *B*, Western blot showing CYP26A1 in WT, RARE1-KO, RARE2-KO, RARE-DKO-1 and RARE-DKO-2 at 0 h and 48 h after RA exposure. GAPDH serves as a loading control, and relative CYP26A1 protein levels are indicated above the blot. *C*, bulk RNA-seq time courses (FPKM) of *Hoxa1* (*left*) and *Hoxb1* (*right*) in WT, RARE-DKO-1 and RARE-DKO-2 at the indicated time points after RA exposure (0, 12, 24, 48 and 72 h). *D*, heatmap summarizing RA uptake/transport and metabolism-associated transcripts at 48 h after RA exposure in WT, RARE-DKO-1 and RARE-DKO-2 (Rep1-Rep3 as indicated). Numbers in tiles indicate FPKM values, and colors indicate row-wise Z-score-normalized expression. *E*-*F*: GO enrichment dot plots for genes differentially expressed in RARE-DKO-1 relative to WT at 48 h after RA exposure: (*E*) downregulated genes and (*F*) upregulated genes. Dot size indicates Gene Count, and dot color indicates −log10(FDR). *G*, UMAP embedding of single-cell RNA-seq profiles collected at 48 h after RA exposure. *Left*, cells are colored by genotype (WT + RA-48 h *versus* RARE-DKO + RA-48 h, as indicated). *Right*, the same embedding colored by annotated cell states. *H*, cell-state composition at 48 h after RA exposure, shown as the percentage of cells assigned to each annotated state for WT, RARE-DKO-1 and RARE-DKO-2. *I*, feature plot of *Cyp26a1* expression projected onto the UMAP embedding at 48 h after RA exposure (*left*; color intensity indicates expression level). *Right*, violin plot comparing *Cyp26a1* expression distributions between WT and RARE-DKO cells. *J*, Violin plots showing expression distributions of *Cyp26a1*, *Cyp26b1*, *Crabp2* and *Stra6* across the annotated cell states at 48 h after RA exposure.

At the global level, RARE-DKO cells displayed widespread differential gene expression compared with WT cells across the RA time course, particularly during the middle and late stages (Fig. S11, *A* and *B*). Genes related to RA homeostasis showed coordinated remodeling at 48 h, involving metabolic enzymes, transporters, binding factors, and multiple *Cyp26* family members (Fig. 7*D*). Similar changes were observed across the 0 to 72 h time course (Fig. S11, *C*–*E*), indicating that RARE-DKO affects broader RA-related transcriptional programs rather than only *Cyp26a1*. GO enrichment analysis further showed that genes downregulated in RARE-DKO cells were enriched for neural-associated developmental processes, including axonogenesis, synaptic organization, and sensory system development (Fig. 7*E*). In contrast, upregulated genes were enriched for developmental patterning, regionalization, and organic anion transport, together with selected spinal cord- and somite-associated processes (Fig. 7*F*). Analyses across additional time points and both RARE-DKO clones showed similar enrichment of neural-associated, sensory, and axon-related processes among downregulated genes, whereas genes involved in transport, retinoid metabolism, and other developmental programs were altered among the upregulated sets (Fig. S12, *A*–*D*). These results indicate that disruption of the enhancer RAREs perturbs the broader RA-responsive transcriptional program beyond the reduction in *Cyp26a1* expression.

To determine how these transcriptional changes were reflected in early cell-state composition, we performed single-cell RNA-seq after 48 h of RA treatment. Integrated analysis resolved multiple early cell states, including undifferentiated ESCs, neural-associated populations, mesenchymal-like cells, endoderm-like cells, and 2C-like cells (Figs. 7*G* and S13*A*). Cell-composition analysis revealed significant differences in cell-state composition in RARE-DKO cells compared with WT cells (Fig. 7*H*). This redistribution was accompanied by reduced *Cyp26a1* expression at the single-cell level (Fig. 7*I*). Genes related to RA homeostasis, including Cyp26 family members and factors associated with RA transport and binding, exhibited state-dependent expression patterns that were altered in RARE-DKO cells (Figs. 7*J* and S13*B*). Lineage-associated scores further illustrated this shift: Pluripotency-associated signatures were elevated in RARE-DKO samples, whereas neural-related signatures were reduced, consistent with the altered cell-state composition (Fig. S13, *C*–*F*). Collectively, these findings demonstrate that the RARE motifs within the enhancer are essential for efficient RA-induced *Cyp26a1* activation. Their loss is accompanied by coordinated remodeling of RA metabolic circuitry, RA-responsive transcriptional programs, and early fate-branch composition.

### Cross-genotype single-cell analysis reveals convergent and distinct differentiation defects across the *Cyp26a1* regulatory axis

To directly compare the consequences of disrupting different components of the *Cyp26a1* regulatory axis, we jointly integrated single-cell transcriptomes from WT, *Cyp26a1*-KO, Enhancer-KO, and RARE-DKO cells after 48 h of RA treatment. The integrated analysis resolved a common set of early differentiation states and revealed both shared and genotype-specific changes in cell-state composition (Fig. S14*A*). Relative to WT cells, all three perturbations shifted cells away from neuroectoderm-associated differentiation, although the magnitude of this effect differed across genotypes. *Cyp26a1*-KO and RARE-DKO cells showed the strongest expansion of undifferentiated ESC and mesenchymal-like populations together with marked depletion of the neuroectoderm-like compartment, whereas Enhancer-KO cells displayed a milder intermediate phenotype (Fig. S14*B*).

Transcriptional program analysis further distinguished the molecular responses associated with each perturbation. WT cells retained stronger RA-target activity, whereas the mutant genotypes showed differential changes in pluripotency-, RA signaling-, cell-cycle-, and mesoderm/mesenchymal-associated programs (Fig. S14*C*). Marker-level analyses within shared cell states also revealed genotype-dependent differences in pluripotency-, neuroectoderm-, mesoderm-, and endoderm-associated transcriptional features (Fig. S14, *C*–*G*). Together, these cross-genotype comparisons show that disruption of *Cyp26a1* or its distal regulatory module produces a convergent shift in RA-guided differentiation while retaining distinct transcriptional consequences that reflect the level of regulatory perturbation.

## Discussion

RA is a dose- and time-sensitive developmental morphogen whose activity depends on the coordination of receptor-mediated transcription with metabolic mechanisms that limit ligand availability 16, 38, 39. Our study identifies an endogenous chromatin mechanism that contributes to this feedback during RA-induced differentiation of mESCs. We show that CYP26A1, a major RA-catabolizing enzyme, is controlled by a highly RA-responsive distal enhancer containing two functional RA response elements (RAREs). RAR/RXR occupancy at these motifs promotes p300 recruitment, local H3K27ac accumulation, and efficient enhancer-promoter communication, thereby supporting full *Cyp26a1* induction (Fig. 8). Genetic disruption of *Cyp26a1*, its distal enhancer, or the enhancer-embedded RAREs consistently altered RA-responsive transcriptional programs and shifted the distribution of early differentiation states. Cross-genotype single-cell analysis further revealed a shared reduction in neuroectoderm-associated differentiation, accompanied to varying degrees by expansion of undifferentiated ESC and mesenchymal-like populations. These findings link cis-regulatory control of RA clearance to the maintenance of appropriate developmental responses to RA.

**Figure 8:**
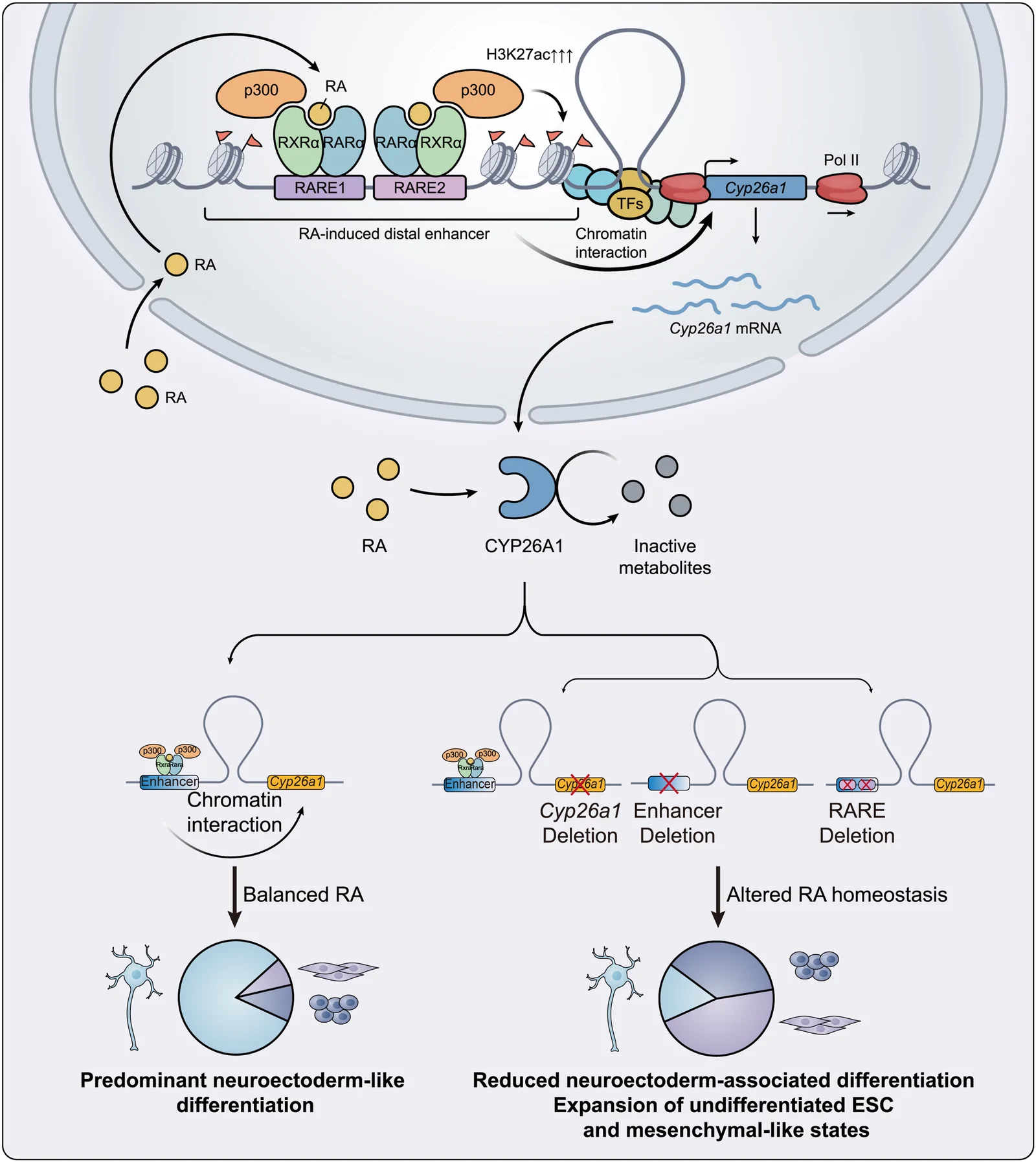
**Model for RA-responsive *Cyp26a1* enhancer-promoter communication in RA homeostasis and early fate balance.** Schematic summarizing the proposed regulatory architecture at the *Cyp26a1* locus during RA exposure. *Top*, RA-bound RARα/RXRα complexes occupy RARE1 and RARE2 within the RA-responsive distal enhancer, recruit p300, promote H3K27ac enrichment, and engage the *Cyp26a1* promoter through chromatin interaction to support RNA polymerase II (Pol II)-associated transcription. The resulting CYP26A1 metabolizes RA into inactive metabolites and contributes to RA homeostasis. *Bottom*, schematic comparison of the differentiation-state distributions associated with WT cells and perturbation of the *Cyp26a1* regulatory axis. In WT cells, intact enhancer-promoter communication and CYP26A1-dependent RA clearance are associated with predominant neuroectoderm-like differentiation. Disruption of *Cyp26a1*, the distal enhancer, or its RAREs is associated with altered RA homeostasis, reduced neuroectoderm-associated differentiation, and increased representation of undifferentiated ESC and mesenchymal-like states.

Our results extend previous studies of the epigenetic regulation of *Cyp26a1*. Previous studies showed that histone deacetylases, including HDAC1, HDAC2, and HDAC3, occupy RARE-containing regulatory regions of *Cyp26a1* in the absence of RA, whereas RA exposure reduces HDAC occupancy and increases H3K27ac (40). Other studies have also implicated repressive chromatin states and, in some cellular contexts, promoter DNA methylation in controlling *Cyp26a1* responsiveness (41). Our findings are consistent with this broader chromatin-based regulation but define an additional level of control at a distal endogenous enhancer. In particular, RAR/RXR loss reduced p300 recruitment and H3K27ac at the enhancer, while pharmacological inhibition of p300 catalytic activity with A-485 decreased H3K27ac and *Cyp26a1* induction without preventing p300 occupancy. These results distinguish coactivator recruitment from its catalytic function and support a model in which p300-mediated acetylation contributes directly to full enhancer activity. H3K27ac should therefore be viewed as one component of a broader RA-responsive chromatin transition that also involves deacetylation, local chromatin state, and three-dimensional enhancer-promoter organization.

The differentiation phenotypes observed here are consistent with, and substantially extend, earlier studies of *Cyp26a1*-deficient ESCs. Previous studies reported that *Cyp26a1* knockout increased intracellular RA, reduced sensitivity to RA-induced proliferation arrest, and decreased the expression of differentiation-associated genes, including neural-associated genes such as *Epha4* and *Nrp1* 42, 43. Our findings extend these observations by resolving the cellular and regulatory basis of the impaired differentiation phenotype. Single-cell transcriptomic analysis shows that loss of *Cyp26a1* reshapes the distribution of early differentiation states, with a reduced neuroectoderm-like compartment and increased representation of undifferentiated and mesenchymal-like populations. By integrating these cell-state changes with time-resolved transcriptomics and targeted disruption of the distal enhancer and its RARE motifs, we further connect defective RA clearance to a defined endogenous cis-regulatory mechanism. Cross-genotype comparisons reveal a shared direction of differentiation disruption across *Cyp26a1*-KO, Enhancer-KO, and RARE-DKO cells while also distinguishing the severity of their effects. This regulatory perspective is consistent with recent evidence that *Aldh1a2*-linked enhancers can modulate RA production in regenerative contexts and extends this principle to the control of RA clearance during pluripotent-cell differentiation (44). Together, these findings support a broader model in which context-specific cis-regulatory elements calibrate both RA production and degradation, thereby shaping the amplitude and developmental consequences of RA signaling.

A small 2-cell-like population was detected across our single-cell datasets. Previous studies have shown that RA can promote a transient 2C-like state characterized by activation of zygotic genome activation-associated genes such as Zscan4 (45). In our data, the relative abundance of 2C-like cells varied among genotypes, with increases in some enhancer- or RARE-disrupted conditions. Because our single-cell analyses capture fixed endpoints, whether these changes reflect direct regulation by the *Cyp26a1* axis or secondary effects of altered RA exposure remains unresolved.

Our genetic dissection revealed a graded regulatory effect across the different perturbations. Enhancer deletion substantially reduced RA-induced *Cyp26a1* transcription and decreased CYP26A1 protein abundance to approximately 50% of WT levels at 48 h, but its effects on cell-state composition were milder than those observed after complete *Cyp26a1* loss or combined RARE deletion. This pattern does not undermine the regulatory significance of the enhancer. Rather, it indicates that transcriptional perturbations do not always scale linearly with protein abundance or phenotypic severity 46, 47, especially in signaling-metabolic circuits with buffering and threshold-like behavior 48, 49. Differences in RARE spacing and local sequence context, including DR1, DR2, and DR3-DR5 elements (direct repeats of receptor-binding half-sites separated by one, two, or three to five nucleotides), may confer distinct regulatory properties and make precise RARE deletion more disruptive to receptor-dependent activation 50, 51. Developmental genes are also often controlled by enhancer ensembles with partially overlapping or temporally distinct activities 52, 53, 54. After deletion of one enhancer, remaining promoter-proximal or distal regulatory elements may partially compensate during RA stimulation, potentially through altered promoter contact frequency or alternative enhancer usage (55). Functional overlap among CYP26 family members may provide an additional layer of compensation. CYP26A1, CYP26B1, and CYP26C1 all metabolize RA, but their developmental functions are only partially redundant because their spatial and temporal expression differs (56). Genetic studies have shown particularly clear cooperation between CYP26A1 and CYP26C1 during embryonic patterning, whereas CYP26B1 also performs distinct tissue-specific functions (57). In our mESC system, *Cyp26b1* and *Cyp26c1* expression changed following disruption of *Cyp26a1* or its regulatory module, consistent with remodeling of the broader RA metabolic network, but these changes were insufficient to prevent the observed differentiation phenotypes. Combinatorial perturbation of CYP26 family members will be required to define the extent of functional compensation in this differentiation context.

Crucially, the regulatory logic of this enhancer module extends beyond early embryogenesis and holds significant clinical relevance for human pathologies. Aberrant cell fate determination and dedifferentiation are common hallmarks of tumorigenesis (58). Consistent with this, our pan-cancer analysis reveals that *CYP26A1* is markedly dysregulated across multiple human malignancies, with its elevated expression significantly correlating with poor disease-free and overall survival in cancers such as kidney renal clear cell carcinoma (Fig. S15, *A*–*E*). Strikingly, epigenomic profiling demonstrates that a distal locus corresponding to the murine *Cyp26a1* enhancer region characterized here, exhibits strong H3K27ac enrichment not only in normal human pluripotent stem cells but also across a broad spectrum of human cancer cell lines, including glioblastoma, lung, and pancreatic cancers. This shared epigenetic landscape strongly suggests that the developmental enhancer module we identified may be hijacked during tumorigenesis to manipulate RA metabolism, thereby sustaining malignant or undifferentiated cell states. This highlights the *Cyp26a1* enhancer-RARE axis as a highly conserved epigenetic vulnerability that bridges embryonic cell fate regulation and oncogenic progression.

In summary, we demonstrate that RA homeostasis during mESC differentiation is actively instructed at the level of enhancer activity and chromatin organization. The unique *Cyp26a1* enhancer-RARE module identified here supports timely induction of metabolic clearance and safeguards appropriate fate-state progression under RA exposure. These findings provide a mechanistic basis for how cis-regulatory control of morphogen metabolism stabilizes developmental signaling, ensures reproducible ESC differentiation, and potentially governs cell fate plasticity in human cancers.

## Experimental procedures

### ESC culture

E14 ESCs (ES-E14TG2a) were cultured in undifferentiated conditions in Dulbecco’s modified Eagle’s medium (DMEM, Gibco) supplemented with 15% fetal bovine serum (FBS, Cat# AusGeneX, FBS500-S), 1× Penicillin-Streptomycin (P/S, 100x, Gibco, 15140-122), 1× nonessential amino acids (NEAA, 100×, Gibco, Cat# 11140-050), 1× GlutaMAX Supplement (100x, Gibco, Cat# 35050061), 50 μM β-mercaptoethanol (Sigma), 1000 U/ml leukemia inhibitory factor (LIF, Sigma, 11710035), and 2i (1 μM PD0325901, MedChemExpress; 3 μM CHIR99021, MedChemExpress). The medium was replaced daily when cells were not passaged. All cells were maintained at 37 °C in a 5% CO2 incubator. Cultured cells were routinely tested for mycoplasma and consistently yielded negative results.

### RA-induced ESC differentiation

For RA-induced ESC differentiation, ESCs were digested using Accutase (Stem Cell, Cat# 07920) and centrifuged at an appropriate speed after 2 to 3 days of culture in complete medium. After centrifugation, cells were resuspended in differentiation medium. The differentiation medium consisted of DMEM supplemented with 15% fetal bovine serum, 1× nonessential amino acids, 1× GlutaMAX Supplement, 50 μM β-mercaptoethanol (Sigma), and 2 μM retinoic acid (RA) (Sigma) at a final concentration. Following plating and counting, cells were passaged at appropriate densities into culture dishes coated with 0.1% gelatin and maintained with daily medium changes.

### Construction of sgRNA plasmids

sgRNA plasmids were constructed using reported protocols (59). Target-specific guide RNAs (sgRNAs) were designed using an online tool (https://portals.broadinstitute.org/gppx/crispick/public). The corresponding *Cyp26a1* and enhancer reference sequences are presented in Table S1. Systematic alignment against the target sequence was performed to screen for sgRNAs matching the target site. Sequence synthesis was outsourced to Shanghai Sangon Biotech Co., Ltd. The sgRNA was ligated into the Cas9-puro vector (pXPR_001) using BsmBI restriction sites (NEB, R0580L). The extracted plasmid was validated *via* Sanger sequencing by GENEWIZ to confirm successful sequence ligation and plasmid construction. The sgRNA sequences used are listed in Table S2.

### CRISPR/Cas9-mediated genome editing in ESCs

Genome editing was performed according to the following methods. The concentration of each successfully constructed plasmid was measured. A mixture of the knockout plasmids at the specified concentrations was prepared and transfected using Lipofectamine 3000 (Life Technologies). After 48 h, cells were selected with 1 μM puromycin (MCE) for 48 h. The selected cells were digested, centrifuged, passaged at an appropriate ratio, and cultured in complete medium for 5 to 7 days. Individual clones were screened and validated *via* genomic DNA PCR (gDNA-PCR), Sanger sequencing, Western blotting, or RT-qPCR. *Cyp26a1* and the *Cyp26a1* distal enhancer were knocked out using a combination of knockout plasmids. Homozygous knockout cell lines were identified *via* gDNA-PCR using sequence-specific primers. The RARE elements were knocked out in multiple steps using two pairs of combination plasmids. Homozygous RARE knockout lines were identified by gDNA-PCR and Sanger sequencing. The Rara/Rxra double-knockout (Rar-DKO) cell line was described previously in our study (12). Table S3 lists the gDNA-PCR primer sequences used for genotyping. All knockout samples were identified by gDNA-PCR and sequenced (Fig. S16, *A*–*C*).

### RNA extraction and qRT-PCR

At different differentiation time points, cell suspensions were digested and centrifuged. Cell pellets were collected and resuspended in TRIzol reagent (Life Technologies) for total RNA extraction. cDNA synthesis was performed using the PrimerScript reverse transcription kit (TaKaRa) with gDNA removal functionality, following the kit's protocol. PCR reactions were performed using Hieff qPCR SYBR Green Master Mix (YEASEN) on a BioRad CFX Connect real-time quantitative fluorescence system. PCR cycling conditions were: 10 min pre-denaturation at 95 °C, followed by 40 cycles (denaturation at 95 °C for 15 s, annealing at 56 °C for 20 s, extension at 72 °C for 20 s). Melt curve data were collected post-amplification. Target gene quantification was normalized using Gapdh as an internal control. Data were analyzed using the comparative Ct (ΔΔCt) method to quantitate gene expression (60). Primer sequences are detailed in Table S4.

### Western blotting analysis

The cell pellet was lysed in cell lysis buffer at 4 °C, and protein concentration was measured using the Pierce BCA Protein Assay Kit (Thermo Scientific). A 10% SDS-PAGE gel was prepared using an SDS-PAGE gel preparation kit (Solarbio) for electrophoresis. Western blotting was performed using the following primary antibodies: (primary antibody) CYP26A1 (primary antibody, Abcam, dilution ratio 1:1000) and GAPDH (primary antibody, Santa Cruz, dilution ratio 1:2000), followed by HRP-labeled secondary antibody (Abcam, Cat# ab6728). After overnight incubation at 4 °C, detection was performed using Luminol HRP substrate (Millipore, Cat# WBKLS0500). Digital images were acquired *via* an automated chemiluminescence imaging system (Cytiva/Amersham ImageQuant 800), and Densitometric quantification was performed using ImageJ software. All raw images are included in the supplementary materials.

### Alkaline phosphatase (AP) staining analysis

After digestion, centrifugation, and resuspension, ESCs were plated at an appropriate density into gelatin-coated 12-well plates and cultured for 5 days. Differentiated E14 cells were seeded at an appropriate density, with differentiation medium replaced daily and samples collected at fixed intervals. *Cyp26a1*-KO samples were similarly seeded at an appropriate density. After 48 h of differentiation medium treatment, samples were collected and matched with corresponding time-point WT samples for comparison. Following media removal, cells were washed 2 to 3 times with PBS and fixed with 4% paraformaldehyde for 2 min. Subsequently, they were incubated with an AP staining kit (System Biosciences) for 20 min and immersed in PBS. Digital images were captured using an Olympus inverted fluorescence microscope. The acquired images were further analyzed using ImageJ software, and AP staining intensity was quantified based on grayscale values. The statistics were calculated using Microsoft Excel and GraphPad Prism 9 software.

### Chromatin immunoprecipitation

A cell pellet of approximately 1 × 10^7^ cells was taken and spin-crosslinked at room temperature with 1% formaldehyde in PBS for 10 min. Subsequently, glycine was added to a final concentration of 125 mM to neutralize the formaldehyde. After incubating for 5 min, samples were centrifuged at 4 °C for 10 min and washed twice with cold PBS. The cell pellets were then collected. The pellet was incubated with lysis buffer containing 100× protease inhibitor mix (Merck, EMD Millipore, Cat# 3446024) on ice for 30 min. Chromatin was fragmented by sonication to an average size of 200 to 500 bp. Fragmentation efficiency was verified by agarose gel electrophoresis or equivalent DNA fragment analysis before immunoprecipitation. Five percent of each sonicated chromatin sample was reserved as input DNA. After pre-clearing, samples were incubated overnight with 7.5 μg each of H3K27ac antibody (abcam, Cat# ab4729), P300 antibody (abcam, Cat# ab275378), RXRα antibody (Proteintech, Cat# 21218-1-AP), RARα antibody (Proteintech, Cat# 10331-1-AP), and IgG antibody as a negative control. Subsequently, 60 μl of protein A/G agarose beads (Thermo Fisher Scientific) were added and incubated at 4 °C for 4 h. The beads were washed three times each with Low Salt Wash Buffer, High Salt Wash Buffer, and LiCl Wash Buffer. Bound chromatin was eluted using DNA elution buffer. Samples were treated with proteinase K at 55 °C for 45 min, followed by reverse cross-linking at 67 °C for 2 h. After reverse cross-linking, DNA was purified using AMPure XP beads. Finally, the concentration was measured for subsequent ChIP-qPCR experiments and analysis. For ChIP-qPCR assays, a chromatin region lacking appreciable H3K27ac or other assayed signal enrichment in WT cells before and after RA treatment was used as the negative control (mm10, chr19: 37,944,163–37,944,295). Primer sequences are detailed in Table S4.

### A-485 treatment

To determine an appropriate working concentration of A-485, WT mESCs were treated with a range of A-485 concentrations in the presence or absence of RA. The concentration was selected based on two criteria: absence of obvious cell death and a clear reduction in global H3K27ac protein levels. Based on these preliminary experiments, 3 μM A-485 was used for subsequent assays. WT mESCs were then cultured under four conditions: vehicle control, A-485 alone, RA alone, or RA combined with A-485. After 48 h of treatment, cells were harvested for subsequent ChIP-qPCR and related downstream analyses.

### Co-IP

Cells treated WT and RAR/RXR-DKO mESCs were lysed in IP lysis buffer. Five percent of the lysate was reserved as input. The remaining cell lysate was first pre-cleared for 2 h at 4 °C using Protein A/G agarose beads. The pre-cleared lysate was then placed on a shaking platform and incubated overnight at 4 °C with P300 antibody (abcam, Cat#ab275378), RXRα antibody (Proteintech, Cat#21218-1-AP), and RARα antibody (Proteintech, Cat#10331-1-AP) or IgG (as a negative control). The next day, protein A-agarose beads were added and incubated at 4 °C for 2 h. The beads were collected by centrifugation, washed four times with IP lysis buffer, and finally placed in SDS sample buffer for boiling. Samples were used for subsequent western blotting analysis.

### scRNA-seq analysis

Single-cell RNA-seq libraries were sequenced on an Illumina platform by LC-BIO Co., Ltd. Raw gene counts were obtained by aligning reads to the mm10 genome (refdata-gex-mm10-2020-A) using CellRanger software (v8.0.1) (10x Genomics). To account for unspliced nuclear transcripts, reads mapping to pre-messenger RNA were also counted. Most subsequent analyses were performed in R using the Seurat package (v4.4.0). Potential doublets were first identified and scored with the Scrublet Python package (v0.2.3). Cells with a doublet score below 0.2 were retained for downstream analysis. We then applied the standard Seurat workflow for quality control and preprocessing. This included filtering out cells that met any of the following criteria (61): fewer than 2000 unique genes detected (62), more than 8000 unique genes detected, or (63) a mitochondrial read percentage exceeding 10%. Following filtering, gene expression counts for each cell were normalized for sequencing depth (using a method analogous to counts per 10,000) and log-transformed. The resulting log-normalized values were then centered and scaled for downstream analysis.

The samples were analyzed jointly within the Seurat framework. First, we selected the top 2500 highly variable genes and performed principal component analysis, retaining the top 30 principal components (PCs). To correct for sample-specific batch effects, we applied batch effect correction using the Harmony algorithm, specifying the individual sample IDs as the batch variable. Based on the Harmony-corrected embeddings, we constructed a shared nearest neighbor (SNN) graph. Cell clustering was then performed using the default graph-based clustering algorithm in the FindClusters function of Seurat. Finally, the integrated cells were visualized in two dimensions using a UMAP (Uniform Manifold Approximation and Projection) embedding calculated with custom parameters.

Cluster annotation was performed manually by integrating two key sources of information. First, we referred to the expression patterns of canonical marker genes, such as those for pluripotency (*Nanog*, *Pou5f1*, *Klf4*, *Zfp42*), 2C-like state (*Zscan4c*, *Zscan4d*, *Zscan4e*, *Gm8994*), neuroectoderm (*Sox1*, *Pax6*, *Zic1*, *Zic2*), and definitive endoderm (*Gata6*, *Gata4*, *Sox17*, *Foxa2*), which were visualized and assessed using Seurat. Second, the biological relevance of each cluster was further evaluated through functional enrichment analysis. Gene Ontology (GO) terms enriched among the highly expressed genes in each cluster were identified using the clusterProfiler (64) package, providing complementary evidence for cell type or state assignment.

To compare transcriptional profiles between conditions, we identified differentially expressed genes between predefined groups (WT *versus* KO) using the FindMarkers method in Seurat. To further assess the enrichment levels of gene sets of interest, we evaluated the activity of biologically relevant pathways or signatures at single-cell resolution using the AddModuleScore function. The average enrichment score per cell cluster was then calculated to represent the overall activity level of each gene set within that cluster.

### Bulk RNA-Seq analysis

RNA libraries were sequenced on the Illumina sequencing platform by LC-BIO Co., Ltd. Raw RNA-Seq reads were quality-checked and trimmed using Fastp (65) to remove low-quality bases and adapter sequences. Cleaned reads were aligned to the mouse reference genome (mm10) (66) with Hisat2. Transcript assembly and quantification were then performed using StringTie, and gene expression levels were estimated as FPKM (fragments per kilobase of transcript per million mapped reads). Gene-level read counts were obtained using the prepDE.py script provided with the StringTie (67) package. Differential expression analysis was carried out using the DESeq2 package in R (68). Genes with an adjusted *p*-value (FDR) < 0.05 and absolute log2 fold change > 1.5 were defined as significantly differentially expressed.

To evaluate the enrichment of gene sets of interest across different samples, we employed the single-sample Gene Set Enrichment Analysis (ssGSEA) algorithm from the GSVA package (69). This method quantifies the activity level of predefined gene signatures within each sample. In a separate analysis, to investigate the temporal dynamics of gene expression in embryonic stem cells (ESCs) following retinoic acid (RA) treatment, we performed soft clustering of time-series data using the Mfuzz R package (70). Genes were grouped into 8 distinct clusters based on their expression patterns over time. The biological functions associated with each gene cluster were subsequently elucidated through Gene Ontology (GO) enrichment analysis using the clusterProfiler (64) package.

### Circular chromosome conformation capture sequencing (4C-seq) data analysis

Analysis of 4C-seq data was performed using the pipe4C pipeline (71). This pipeline automates the primary steps: demultiplexing sequencing reads based on primer sequences, trimming reads according to restriction enzyme recognition sites, aligning reads to the reference genome (mm10) using Bowtie2, and assigning read counts to restriction fragment ends (72). The data were subsequently normalized and smoothed using a sliding-window approach to generate fragment-level interaction profiles for downstream analysis. Specific interactions between the Cyp26a1 promoter and its candidate enhancer region (chr19: 37,786,499–37,794,133) were identified by applying thresholds of > 10 raw read counts and a normalized enrichment score > 4. The resulting interactions were visualized using the WashU Epigenome Browser (73).

### Hi-C analysis

The Hi-C analysis in this study employed published data (74). HiC-Pro (75) was used for Hi-C reads mapping. The clean Hi-C reads of ESCs were aligned to the mouse reference genome (mm10) after removing the adapter. Following processing with HiC-Pro and Juicer software, valid pairs of Hi-C data were used to create interaction matrices at a bin size of 50 kb for further analysis. Subsequently, topologically associating domains (TADs) were identified using the hicFindTADs function from the HiCExplorer (76) toolkit with the following parameters: −minDepth 60000, −maxDepth 600000, −step 10000, −thresholdComparisons 0.05, −delta 0.01, −correctForMultipleTesting “fdr”, and −p 20. Prior to TAD calling, interaction matrices obtained from HiC-Pro were converted to.h5 format using the hicConvertFormat utility. Finally, identified TADs were visualized with the hicPlotTADs function.

### CUT&Tag-seq analysis

Raw CUT&Tag-seq reads were quality-checked and trimmed using Fastp (65) to remove low-quality bases and adapter sequences. Cleaned reads were aligned to the mouse reference genome (mm10) using the BWA-MEM algorithm (77). Using SAMtools, we filtered aligned reads to remove those with a mapping quality (MAPQ) score below 30 and PCR duplicates. The MACS2 peak caller (78) was used to identify narrow peaks (macs2 callpeak -t <input file> -f BAM -n < output peak file > -B -q 0.05 -g mm10). The CUT&Tag data were visualized using the integrative genomics viewer (IGV) (79).

### Pan-cancer expression and survival analysis

The pan-cancer differential expression of *CYP26A1* between tumor tissues and adjacent normal tissues was analyzed using the GEPIA 2 (http://gepia2.cancer-pku.cn/#index) and GEPIA 3 (https://gepia3.bioinfoliu.com/) web servers, which integrate RNA sequencing data from the TCGA and GTEx projects (80). Gene expression levels were normalized as log2(TPM + 1). For survival analysis, overall survival (OS), and disease-free survival (DFS) were evaluated across multiple cancer types. Patients were stratified into high- and low-expression groups based on the median *CYP26A1* expression level. Kaplan-Meier survival curves were generated, and statistical significance was assessed using the log-rank test. Hazard ratios (HR) and corresponding *p*-values were also calculated.

### Epigenetic landscape and enhancer region visualization

To explore the potential regulatory mechanisms of *CYP26A1*, the epigenetic signal tracks at the *CYP26A1* genomic locus (hg38, chr10:93,049,734–93,222,579) were evaluated. Specifically, H3K27ac ChIP-seq data from various human cancer cell lines and normal/stem cells were downloaded and analyzed using the Cistrome Data Browser (http://cistrome.org/db/#/) to identify putative enhancer regions (81).

### Dataset availability

We acknowledge the laboratories that generated the publicly available data used in this study. A list of published datasets utilized in this research is detailed in Table S5. These published datasets can be downloaded and analyzed *via* an online tool (http://3dgenome.fsm.northwestern.edu/and www.ncbi.nlm.nih.gov/sra/). The sequencing data generated in this study have been deposited in CNSA with accession number CNP0008840 (http://db.cngb.org/cnsa/project/CNP0008840_bd54e23c/reviewlink/) (82). The data underlying this article are available in the Gene Expression Omnibus (GEO) at https://www.ncbi.nlm.nih.gov/geo/. The publicly accessible 4C-seq data include GSM6911364 (4c-seq-Rep2-WT-Cyp26a1-Promoter), GSM6911365 (4c-seq-Rep1-WT-Cyp26a1-Promoter), GSM6911370 (4c-seq-Rep1-DKO-Cyp26a1-Promoter), and GSM6911371 (4c-seq-Rep2-DKO-Cyp26a1-Promoter). The CUT&Tag and scRNA-seq datasets have been deposited and are availabled which include GSM8504594 (RARX-WT-RA0h-H3K27ac-Cut/tag), GSM8504595 (RARX-WT-RA24h-H3K27ac-Cut/tag), GSM8504596 (RARX-KO-RA24h-H3K27ac-Cut/tag), GSM8504597 (WT-RA24h-RARA-Cut/tag), GSM8504598 (WT-RA24h-RXRA-Cut/tag), and GSM8505030 (ESC-RA24h-scRNA). All data will be made fully publicly available upon publication.

### Statistical analysis

The statistics were calculated using Microsoft Excel and GraphPad Prism 9 software. Statistical tests and replicates for the experiment are presented in the figure legends. Statistically significant *p* values are indicated in the figure legends as follows: ∗*p* < 0.05, ∗∗*p* < 0.01, ∗∗∗*p* < 0.001.

## Data and materials availability

All data needed to evaluate the conclusions in the paper are present in the paper and the Supplementary Materials. Additional data related to this paper may be requested from the authors.

## Supporting information

This article contains supporting information.

## Conflict of interest

The authors declare that they have no conflicts of interest with the contents of this article.

## References

[1] Hamazaki N., Yang W., Kubo C.A., Qiu C., Martin B.K., Garge R.K. (2024). Retinoic acid induces human gastruloids with posterior embryo-like structures. Nat. Cell Biol..

[2] Liu T., He W., Zhong Z., Lu C., Wu L., Wang Z. (2025). The circadian clock orchestrates spermatogonial differentiation and fertilization by regulating retinoic acid signaling in vertebrates. Natl. Sci. Rev..

[3] Maden M. (2002). Retinoid signalling in the development of the central nervous system. Nat. Rev. Neurosci..

[4] Ferreira R., Napoli J., Enver T., Bernardino L., Ferreira L. (2020). Advances and challenges in retinoid delivery systems in regenerative and therapeutic medicine. Nat. Commun..

[5] Tambones I., Sagar A., Vankova P., Loginov D., Carivenc C., Rochel N. (2025). New structural insights into the control of the retinoic acid receptors RAR/RXR by DNA, ligands, and transcriptional coregulators. Nucleic Acids Res..

[6] Clagett-Dame M., DeLuca H.F. (2002). The role of vitamin A in mammalian reproduction and embryonic development. Annu. Rev. Nutr..

[7] See A.W., Kaiser M.E., White J.C., Clagett-Dame M. (2008). A nutritional model of late embryonic vitamin A deficiency produces defects in organogenesis at a high penetrance and reveals new roles for the vitamin in skeletal development. Dev. Biol..

[8] Ghyselinck N.B., Duester G. (2019). Retinoic Acid Signaling Pathways. Development.

[9] Meshorer E., Misteli T. (2006). Chromatin in pluripotent embryonic stem cells and differentiation. Nat. Rev. Mol. Cell Biol..

[10] Martello G., Smith A. (2014). The nature of embryonic stem cells. Annu. Rev. Cell Dev Biol..

[11] Su G., Guo D., Chen J., Liu M., Zheng J., Wang W. (2019). A distal enhancer maintaining Hoxa1 expression orchestrates retinoic acid-induced early ESCs differentiation. Nucleic Acids Res..

[12] Su G., Wang W., Zhao X., Chen J., Zheng J., Liu M. (2021). Enhancer architecture-dependent multilayered transcriptional regulation orchestrates RA signaling-induced early lineage differentiation of ESCs. Nucleic Acids Res..

[13] Su G., Wang W., Chen J., Liu M., Zheng J., Guo D. (2021). CTCF-binding element regulates ESC differentiation via orchestrating long-range chromatin interaction between enhancers and HoxA. J. Biol. Chem..

[14] Yin Y., Yan P., Lu J., Song G., Zhu Y., Li Z. (2015). Opposing roles for the lncRNA haunt and its genomic locus in regulating HOXA gene activation during embryonic stem cell differentiation. Cell Stem Cell.

[15] Pennimpede T., Cameron D.A., MacLean G.A., Li H., Abu-Abed S., Petkovich M. (2010). The role of CYP26 enzymes in defining appropriate retinoic acid exposure during embryogenesis. Birth Defects Res. A. Clin. Mol. Teratol..

[16] Abu-Abed S., Dollé P., Metzger D., Beckett B., Chambon P., Petkovich M. (2001). The retinoic acid-metabolizing enzyme, CYP26A1, is essential for normal hindbrain patterning, vertebral identity, and development of posterior structures. Genes Dev..

[17] Sakai Y., Meno C., Fujii H., Nishino J., Shiratori H., Saijoh Y. (2001). The retinoic acid-inactivating enzyme CYP26 is essential for establishing an uneven distribution of retinoic acid along the anterio-posterior axis within the mouse embryo. Genes Dev..

[18] Zabidi M.A., Stark A. (2016). Regulatory enhancer-core-promoter communication via transcription factors and cofactors. Trends Genet..

[19] Su G., Chen B., Song Y., Yin Q., Wang W., Zhao X. (2025). Klf5-adjacent super-enhancer functions as a 3D genome structure-dependent transcriptional driver to safeguard ESC identity. Nat. Commun..

[20] Bi J., Mo W., Liu M., Song Y., Xiao Q., Fan S. (2025). Systematic decoding of functional enhancer connectomes and risk variants in human glioma. Nat. Cell Biol..

[21] Sabari B.R., Dall’Agnese A., Boija A., Klein I.A., Coffey E.L., Shrinivas K. (2018). Coactivator condensation at super-enhancers links phase separation and gene control. Science.

[22] Simandi Z., Horvath A., Wright L.C., Cuaranta-Monroy I., De Luca I., Karolyi K. (2016). OCT4 acts as an integrator of pluripotency and signal-induced differentiation. Mol. Cell.

[23] Kashyap V., Gudas L.J. (2010). Epigenetic regulatory mechanisms distinguish retinoic acid-mediated transcriptional responses in stem cells and fibroblasts. J. Biol. Chem..

[24] Bedois A.M.H., Parker H.J., Price A.J., Morrison J.A., Bronner M.E., Krumlauf R. (2024). Sea lamprey enlightens the origin of the coupling of retinoic acid signaling to vertebrate hindbrain segmentation. Nat. Commun..

[25] Ono K., Keller J., López Ramírez O., González Garrido A., Zobeiri O.A., Chang H.H.V. (2020). Retinoic acid degradation shapes zonal development of vestibular organs and sensitivity to transient linear accelerations. Nat. Commun..

[26] Beccari L., Moris N., Girgin M., Turner D.A., Baillie-Johnson P., Cossy A.C. (2018). Multi-axial self-organization properties of mouse embryonic stem cells into gastruloids. Nature.

[27] Pattison J.M., Melo S.P., Piekos S.N., Torkelson J.L., Bashkirova E., Mumbach M.R. (2018). Retinoic acid and BMP4 cooperate with p63 to alter chromatin dynamics during surface epithelial commitment. Nat. Genet..

[28] Brown C.C., Esterhazy D., Sarde A., London M., Pullabhatla V., Osma-Garcia I. (2015). Retinoic acid is essential for Th1 cell lineage stability and prevents transition to a Th17 cell program. Immunity.

[29] Andersson R., Gebhard C., Miguel-Escalada I., Hoof I., Bornholdt J., Boyd M. (2014). An atlas of active enhancers across human cell types and tissues. Nature.

[30] Weake V.M., Workman J.L. (2010). Inducible gene expression: diverse regulatory mechanisms. Nat. Rev. Genet..

[31] Thomas H.F., Kotova E., Jayaram S., Pilz A., Romeike M., Lackner A. (2021). Temporal dissection of an enhancer cluster reveals distinct temporal and functional contributions of individual elements. Mol. Cell.

[32] Malik S., Roeder R.G. (2010). The metazoan Mediator co-activator complex as an integrative hub for transcriptional regulation. Nat. Rev. Genet..

[33] Hilton I.B., D'Ippolito A.M., Vockley C.M., Thakore P.I., Crawford G.E., Reddy T.E. (2015). Epigenome editing by a CRISPR-Cas9-based acetyltransferase activates genes from promoters and enhancers. Nat. Biotechnol..

[34] Kearns N.A., Pham H., Tabak B., Genga R.M., Silverstein N.J., Garber M. (2015). Functional annotation of native enhancers with a Cas9-histone demethylase fusion. Nat. Methods.

[35] Huang P., Chandra V., Rastinejad F. (2014). Retinoic acid actions through mammalian nuclear receptors. Chem. Rev..

[36] Giannì M., Parrella E., Raska I., Gaillard E., Nigro E.A., Gaudon C. (2006). P38MAPK-dependent phosphorylation and degradation of SRC-3/AIB1 and RARalpha-mediated transcription. EMBO J..

[37] Lee S., Lee B., Lee J.W., Lee S.K. (2009). Retinoid signaling and neurogenin2 function are coupled for the specification of spinal motor neurons through a chromatin modifier CBP. Neuron.

[38] Shimozono S., Iimura T., Kitaguchi T., Higashijima S., Miyawaki A. (2013). Visualization of an endogenous retinoic acid gradient across embryonic development. Nature.

[39] Diez del Corral R., Olivera-Martinez I., Goriely A., Gale E., Maden M., Storey K. (2003). Opposing FGF and retinoid pathways control ventral neural pattern, neuronal differentiation, and segmentation during body axis extension. Neuron.

[40] Urvalek A.M., Gudas L.J. (2014). Retinoic acid and histone deacetylases regulate epigenetic changes in embryonic stem cells. J. Biol. Chem..

[41] Zuchegna C., Aceto F., Bertoni A., Romano A., Perillo B., Laccetti P. (2014). Mechanism of retinoic acid-induced transcription: histone code, DNA oxidation and formation of chromatin loops. Nucleic Acids Res..

[42] Zhang Y., Zolfaghari R., Ross A.C. (2010). Multiple retinoic acid response elements cooperate to enhance the inducibility of CYP26A1 gene expression in liver. Gene.

[43] Langton S., Gudas L.J. (2008). CYP26A1 knockout embryonic stem cells exhibit reduced differentiation and growth arrest in response to retinoic acid. Dev. Biol..

[44] Lin W., Jia X., Shi X., He Q., Zhang P., Zhang X. (2025). Reactivation of mammalian regeneration by turning on an evolutionarily disabled genetic switch. Science.

[45] Iturbide A., Ruiz Tejada Segura M.L., Noll C., Schorpp K., Rothenaigner I., Ruiz-Morales E.R. (2021). Retinoic acid signaling is critical during the totipotency window in early mammalian development. Nat. Struct. Mol. Biol..

[46] Zhao B.S., Roundtree I.A., He C. (2017). Post-transcriptional gene regulation by mRNA modifications. Nat. Rev. Mol. Cell Biol..

[47] Swain P.S. (2004). Efficient attenuation of stochasticity in gene expression through post-transcriptional control. J. Mol. Biol..

[48] Halbeisen R.E., Gerber A.P. (2009). Stress-dependent coordination of transcriptome and translatome in yeast. Plos Biol..

[49] Rambout X., Maquat L.E. (2024). Nuclear mRNA decay: regulatory networks that control gene expression. Nat. Rev. Genet..

[50] Cunningham T.J., Duester G. (2015). Mechanisms of retinoic acid signalling and its roles in organ and limb development. Nat. Rev. Mol. Cell Biol..

[51] Evans R.M., Mangelsdorf D.J. (2014). Nuclear receptors, RXR, and the Big Bang. Cell.

[52] Spitz F., Furlong E.E. (2012). Transcription factors: from enhancer binding to developmental control. Nat. Rev. Genet..

[53] Kvon E.Z., Kazmar T., Stampfel G., Yáñez-Cuna J.O., Pagani M., Schernhuber K. (2014). Genome-scale functional characterization of Drosophila developmental enhancers in vivo. Nature.

[54] Frankel N., Davis G.K., Vargas D., Wang S., Payre F., Stern D.L. (2010). Phenotypic robustness conferred by apparently redundant transcriptional enhancers. Nature.

[55] Long H.K., Prescott S.L., Wysocka J. (2016). Ever-Changing landscapes: transcriptional enhancers in development and evolution. Cell.

[56] Isoherranen N., Zhong G. (2019). Biochemical and physiological importance of the CYP26 retinoic acid hydroxylases. Pharmacol. Ther..

[57] Thatcher J.E., Isoherranen N. (2009). The role of CYP26 enzymes in retinoic acid clearance. Expert Opin. Drug Metab. Toxicol..

[58] Hanahan D. (2022). Hallmarks of cancer: new dimensions. Cancer Discov..

[59] Shalem O., Sanjana N.E., Hartenian E., Shi X., Scott D.A., Mikkelson T. (2014). Genome-scale CRISPR-Cas9 knockout screening in human cells. Science.

[60] Schmittgen T.D., Livak K.J. (2008). Analyzing real-time PCR data by the comparative C(T) method. Nat. Protoc..

[61] Hao Y., Hao S., Andersen-Nissen E., Mauck W.M., Zheng S., Butler A. (2021). Integrated analysis of multimodal single-cell data. Cell.

[62] Wolock S.L., Lopez R., Klein A.M. (2019). Scrublet: computational identification of cell doublets in single-cell transcriptomic data. Cell Syst..

[63] Korsunsky I., Millard N., Fan J., Slowikowski K., Zhang F., Wei K. (2019). Fast, sensitive and accurate integration of single-cell data with Harmony. Nat. Methods.

[64] Yu G., Wang L.G., Han Y., He Q.Y. (2012). clusterProfiler: an R package for comparing biological themes among gene clusters. Omics.

[65] Chen S., Zhou Y., Chen Y., Gu J. (2018). fastp: an ultra-fast all-in-one FASTQ preprocessor. Bioinformatics.

[66] Kim D., Paggi J.M., Park C., Bennett C., Salzberg S.L. (2019). Graph-based genome alignment and genotyping with HISAT2 and HISAT-genotype. Nat. Biotechnol..

[67] Pertea M., Pertea G.M., Antonescu C.M., Chang T.C., Mendell J.T., Salzberg S.L. (2015). StringTie enables improved reconstruction of a transcriptome from RNA-seq reads. Nat. Biotechnol..

[68] Love M.I., Huber W., Anders S. (2014). Moderated estimation of fold change and dispersion for RNA-seq data with DESeq2. Genome Biol..

[69] Hänzelmann S., Castelo R., Guinney J. (2013). GSVA: gene set variation analysis for microarray and RNA-seq data. BMC Bioinformatics.

[70] Kumar L., M E.F. (2007). Mfuzz: a software package for soft clustering of microarray data. Bioinformation.

[71] Krijger P.H.L., Geeven G., Bianchi V., Hilvering C.R.E., de Laat W. (2020). 4C-seq from beginning to end: A detailed protocol for sample preparation and data analysis. Methods.

[72] Langmead B., Wilks C., Antonescu V., Charles R. (2019). Scaling read aligners to hundreds of threads on general-purpose processors. Bioinformatics.

[73] Seng C., Liu S., Zhang W., Zhuo X., Li D., Wang T. (2025). WashU Epigenome Browser update 2025. Nucleic Acids Res..

[74] Yan J., Chen S.A., Local A., Liu T., Qiu Y., Dorighi K.M. (2018). Histone H3 lysine 4 monomethylation modulates long-range chromatin interactions at enhancers. Cell Res..

[75] Servant N., Varoquaux N., Lajoie B.R., Viara E., Chen C.J., Vert J.P. (2015). HiC-Pro: an optimized and flexible pipeline for Hi-C data processing. Genome Biol..

[76] Wolff J., Rabbani L., Gilsbach R., Richard G., Manke T., Backofen R. (2020). Galaxy HiCExplorer 3: a web server for reproducible Hi-C, capture Hi-C and single-cell Hi-C data analysis, quality control and visualization. Nucleic Acids Res..

[77] Li H., Durbin R. (2010). Fast and accurate long-read alignment with Burrows-Wheeler transform. Bioinformatics.

[78] Zhang Y., Liu T., Meyer C.A., Eeckhoute J., Johnson D.S., Bernstein B.E. (2008). Model-based analysis of ChIP-Seq (MACS). Genome Biol..

[79] Robinson J.T., Thorvaldsdottir H., Turner D., Mesirov J.P. (2023). Igv.js: an embeddable JavaScript implementation of the Integrative Genomics Viewer (IGV). Bioinformatics.

[80] Tang Z., Kang B., Li C., Chen T., Zhang Z. (2019). GEPIA2: an enhanced web server for large-scale expression profiling and interactive analysis. Nucleic Acids Res..

[81] Taing L., Dandawate A., L'Yi S., Gehlenborg N., Brown M., Meyer C.A. (2024). Cistrome Data Browser: integrated search, analysis and visualization of chromatin data. Nucleic Acids Res..

[82] Wang W., Tan C., Li L., Li X., Zhang L., Li X. (2025). The China National GeneBank Sequence Archive (CNSA) 2024 update. Hortic. Res..

