# Supplementary material for: An endogenous retinoic acid response element enhancer controls Cyp26a1-mediated retinoic acid clearance during embryonic stem cell differentiation
Source: J Biol Chem. 2026 Sep 29;302(11):113613. doi: 10.1016/j.jbc.2026.113613 (PMC13738024; doi:10.1016/j.jbc.2026.113613)
Supplement: Supplementary file 1 [file mmc1.pdf]

Supplementary Figure 1

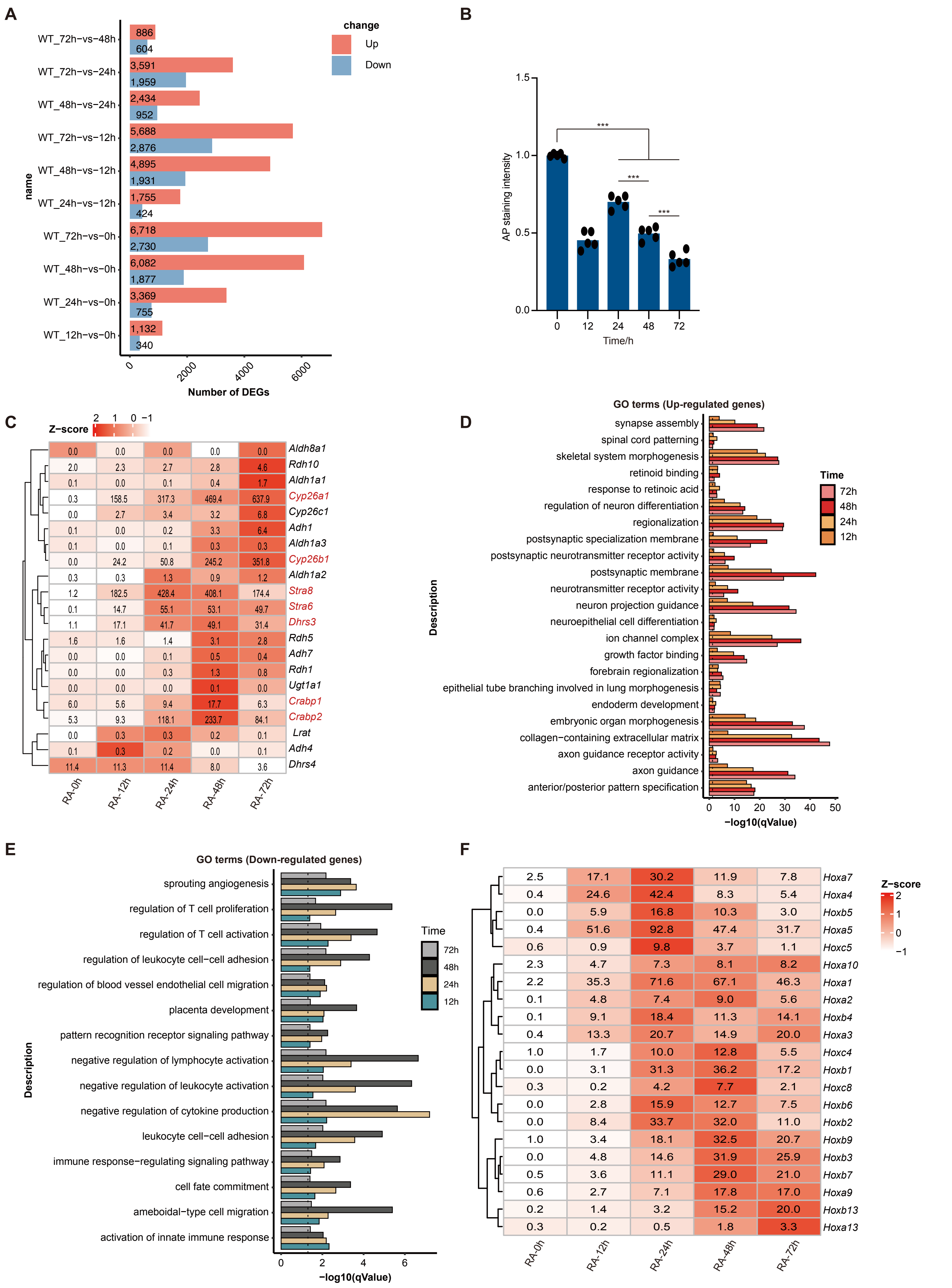

Supplementary Fig. 1.

Global overview of WT RA time-course transcriptomes and developmental gene programs

A: Numbers of DEGs identified across the indicated pairwise comparisons along the RA time course in WT cells. Bars are partitioned into upregulated (pink) and downregulated (blue) genes; numbers indicate DEG counts for each direction.

B: Quantification of AP staining intensity in WT cells at the indicated time points during RA-induced differentiation. Bars indicate the mean, and symbols represent individual replicates. Statistical comparisons are indicated by brackets; ***$P < 0.001$.

C: Heatmap of RA uptake/transport and metabolism-associated transcripts across the WT RA time course (RA-0 h, RA-12 h, RA-24 h, RA-48 h and RA-72 h). Values in tiles indicate expression levels as plotted; colors represent row-wise Z-scores.

D: GO enrichment results for upregulated DEGs at each time point (12 h, 24 h, 48 h and 72 h relative to 0 h). Bar length indicates $-\log10(\mathrm{qValue})$, where qValue denotes multiple-testing–adjusted significance.

E: GO enrichment results for downregulated DEGs at each time point.

F: Heatmap of *Hox* family transcripts across the WT RA time course (0, 12, 24, 48 and 72 h). Values in tiles indicate expression levels, and colors represent row-wise Z-scores.

Supplementary Figure 2

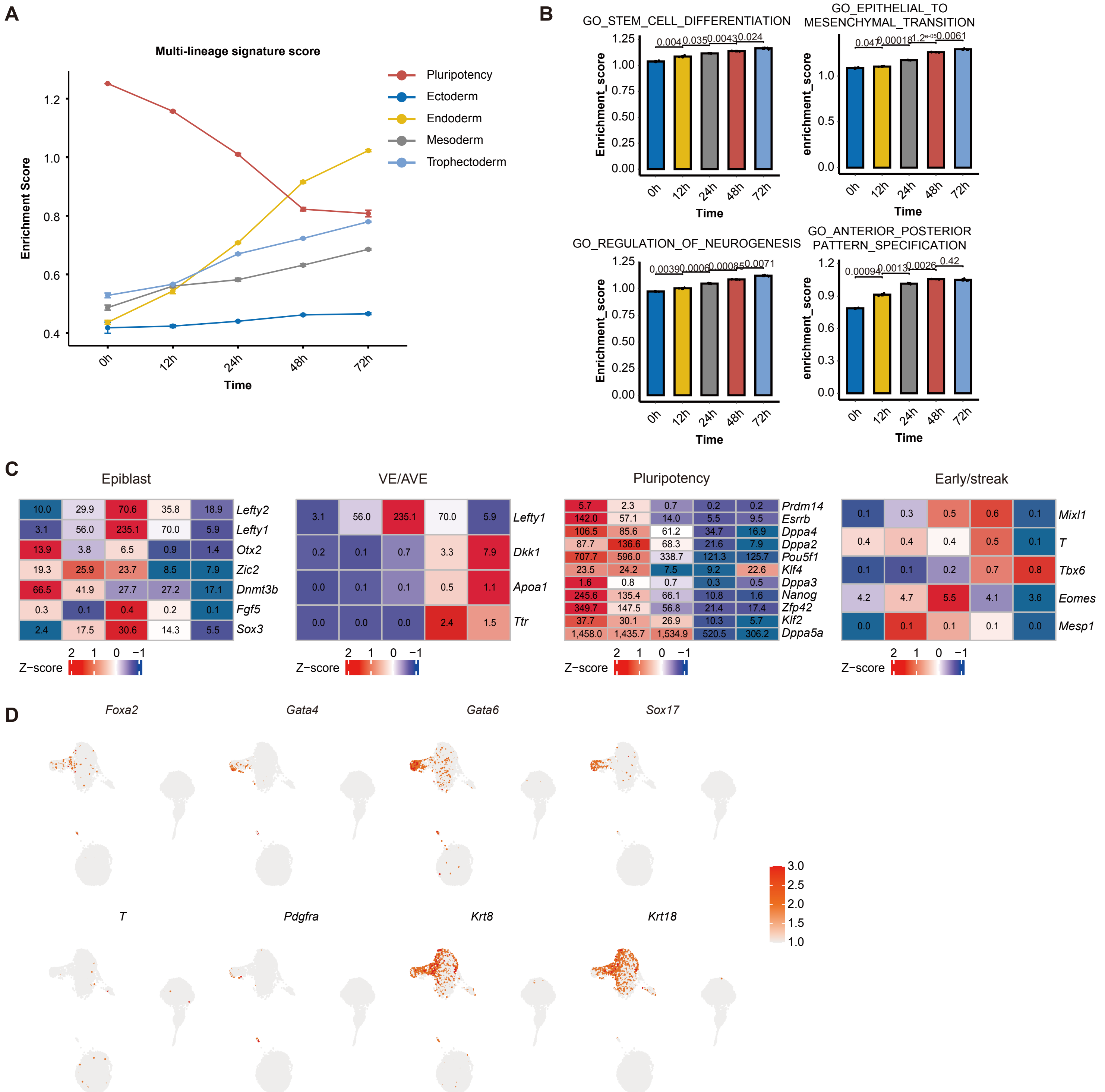

Supplementary Fig. 2.

Developmental-program signature scoring and representative marker expression during RA treatment

A: Developmental-program signature scores across the RA time course (0, 12, 24, 48 and 72 h). Lines denote enrichment scores for the indicated signatures.

B: Bar plots showing enrichment scores for selected GO-BP terms at each time point (0, 12, 24, 48 and 72 h). Bar colors correspond to time points as labelled; nominal P values are shown above bars.

C: Heatmaps showing bulk RNA-seq expression levels of representative marker genes for the indicated developmental programs (Epiblast, VE/AVE, Pluripotency and Early/streak) across the RA time course (columns: 0, 12, 24, 48 and 72 h).

D: Feature plots showing expression of selected endoderm-associated markers (*Foxa2*, *Gata4*, *Gata6* and *Sox17*), primitive streak/mesoderm-associated markers (*T* and *Pdgfra*), and epithelial-associated markers (*Krt8* and *Krt18*) in the single-cell RNA-seq dataset.

Supplementary Figure 3

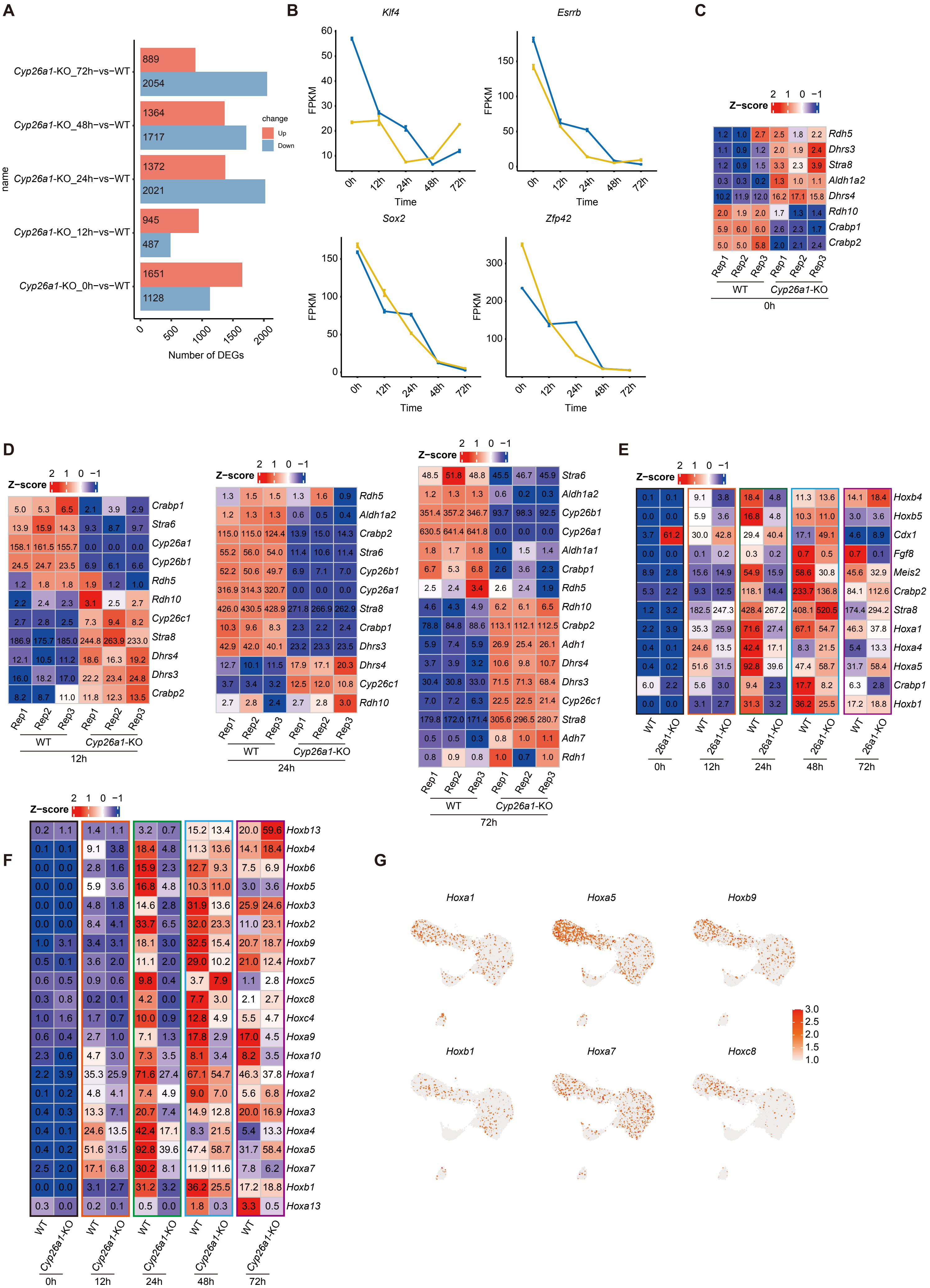

Supplementary Fig. 3.

Time-resolved transcriptional changes and RA-responsive and Hox gene expression in *Cyp26a1*-KO cells

A: Numbers of DEGs identified by bulk RNA-seq when comparing *Cyp26a1*-KO to WT at each indicated time point after RA exposure (0, 12, 24, 48 and 72 h). Bars are partitioned into upregulated (red) and downregulated (blue) genes.

B: Bulk RNA-seq expression trajectories (FPKM) of pluripotency-associated transcription factors across the RA time course in WT (yellow) and *Cyp26a1*-KO (blue).

C: Heatmap of selected RA metabolism/transport-related transcripts at 0 h in WT and *Cyp26a1*-KO (Rep1-Rep3). Numbers in tiles denote expression values as plotted; colors indicate row-wise Z-scores.

D: Heatmaps of RA metabolism/transport-related transcripts at 12 h (left), 24 h (middle) and 72 h (right) in WT and *Cyp26a1*-KO (Rep1-Rep3). Numbers in tiles indicate expression values, and colors represent row-wise Z-scores.

E: Heatmap summarizing bulk RNA-seq expression (values in tiles) of selected RA-responsive and axial-patterning–associated genes across the RA time course (0–72 h) in WT and *Cyp26a1*-KO. Colors indicate row-wise Z-score-normalized expression.

F: Heatmap summarizing bulk RNA-seq expression (values in tiles) of representative *Hox* family transcripts across the RA time course (0-72 h) in WT and *Cyp26a1*-KO. Colors indicate row-wise Z-score-normalized expression.

G: Feature plots showing expression of representative *Hox* genes projected onto the UMAP embedding of single-cell transcriptomes.

Supplementary Figure 4

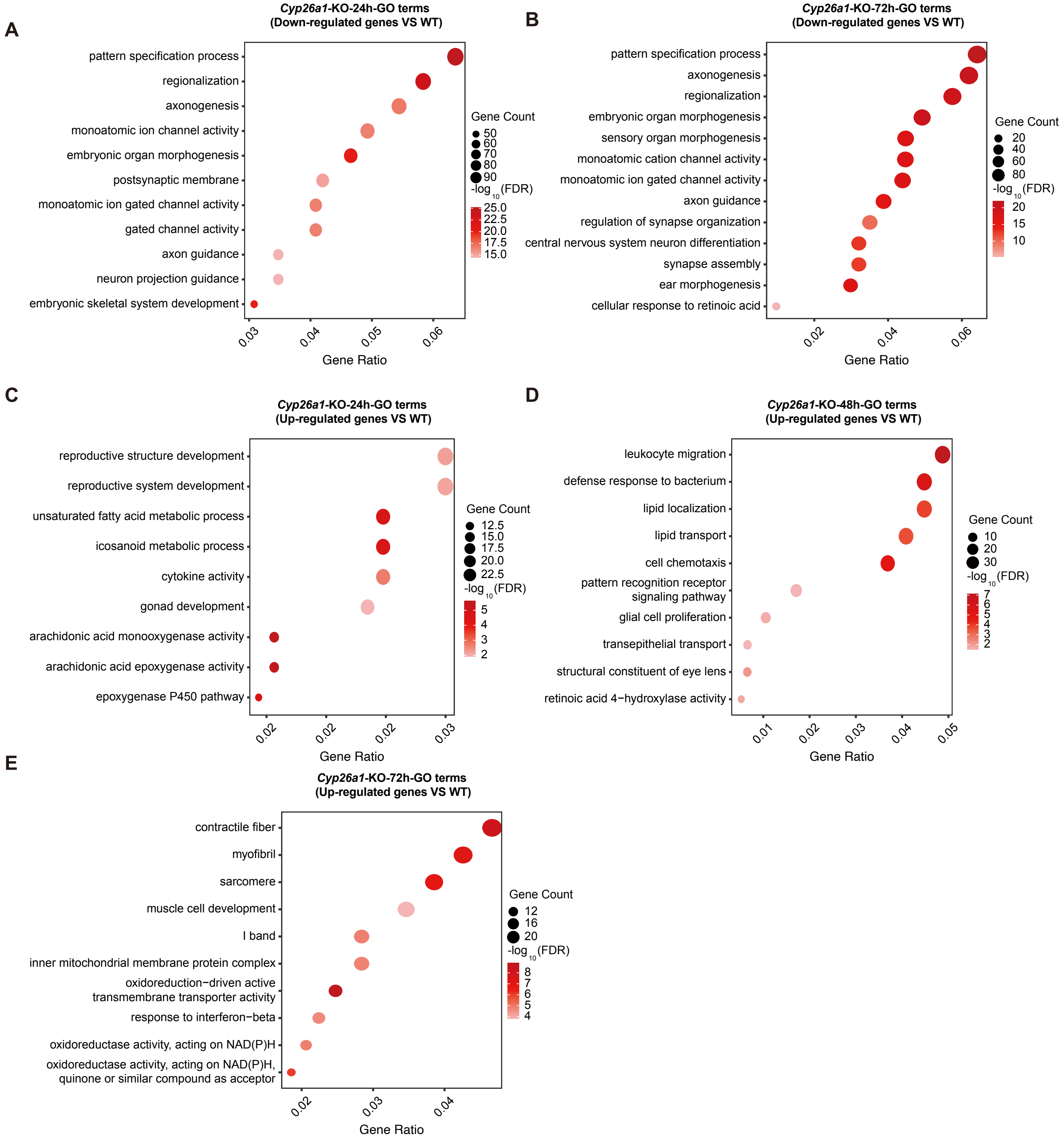

Supplementary Fig. 4.

GO enrichment analysis of differentially expressed genes in *Cyp26a1*-KO cells

A-E: GO enrichment dot plots for differentially expressed genes (DEGs) in *Cyp26a1*-KO relative to WT at the indicated time points after RA exposure: (**A**) 24 h, downregulated genes; (**B**) 72 h, downregulated genes; (**C**) 24 h, upregulated genes; (**D**) 48 h, upregulated genes; (**E**) 72 h, upregulated genes. Dot size indicates Gene Count, and dot color indicates −log10(FDR).

Supplementary Figure 5

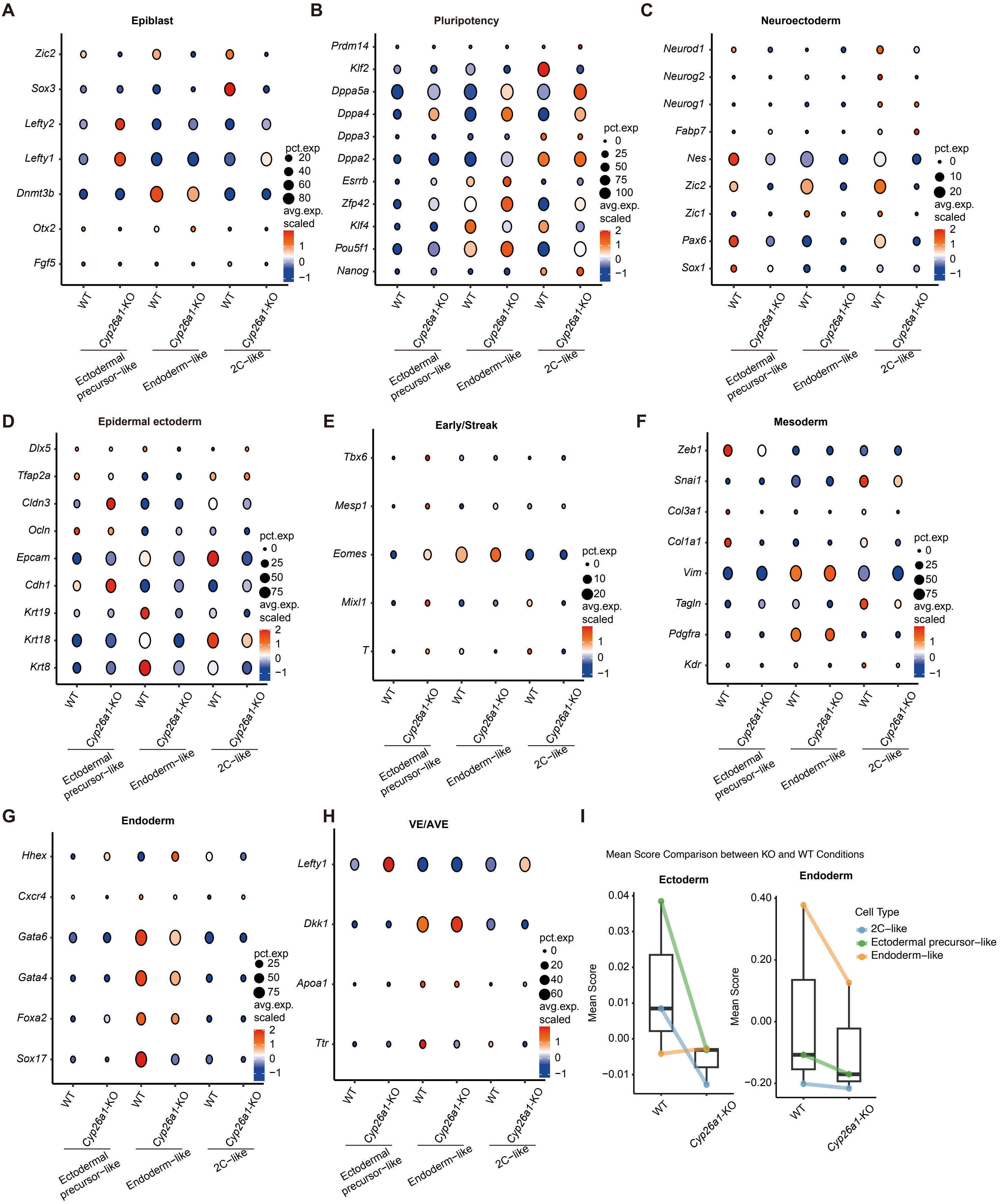

Supplementary Fig. 5.

Single-cell-resolved developmental-state markers and signature-score comparisons between WT and *Cyp26a1*-KO cells

A-H: Dot plots summarizing expression of selected marker genes in WT and *Cyp26a1*-KO cells within the ectodermal precursor-like, endoderm-like and 2C-like populations, as indicated on the x-axis. Dot size indicates the fraction of cells with detectable expression (pct.exp), and dot color indicates scaled average expression (blue, low; red, high). (**A**) epiblast-associated genes; (**B**) pluripotency-associated genes; (**C**) neuroectoderm-associated genes; (**D**) epidermal ectoderm-associated genes; (**E**) early/streak-associated genes; (**F**) mesoderm-associated genes; (**G**) endoderm-associated genes; (**H**) VE/AVE-associated genes.

I: Comparison of ectoderm and endoderm signature scores between WT and *Cyp26a1*-KO. Box plots show the distribution of per-cell gene-set scores for each genotype (WT versus *Cyp26a1*-KO), and colored lines indicate the mean score for each annotated cell state (2C-like, ectodermal precursor-like and endoderm-like). Centre line, median; box, interquartile range; whiskers extend to the most extreme values within 1.5× the interquartile range.

Supplementary Figure 6

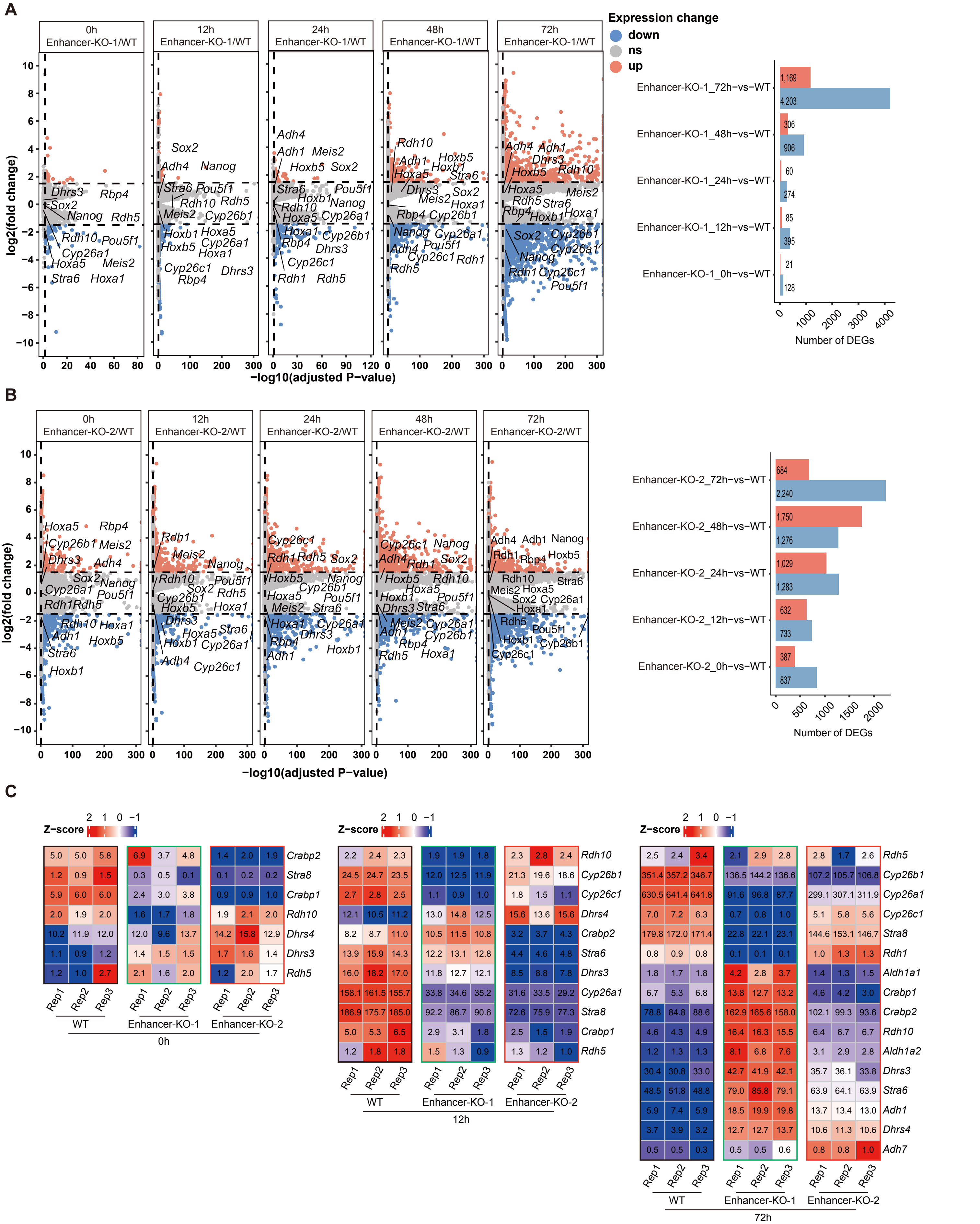

Supplementary Fig. 6.

Time-course differential expression and RA-related transcriptional changes in Enhancer-KO clones

A: Volcano plots showing differential gene expression for Enhancer-KO-1 versus WT at 0, 12, 24, 48 and 72 h after RA exposure (as indicated). Points are colored by expression change (upregulated, red; downregulated, blue; not significant, grey). Right, bar plots summarizing the number of DEGs at each time point, partitioned into upregulated (red) and downregulated (blue) genes; numbers within bars denote gene counts.

B: Volcano plots and DEG counts for Enhancer-KO-2 versus WT at 0, 12, 24, 48 and 72 h after RA exposure.

C: Heatmaps of selected RA uptake/transport and metabolism-associated transcripts at 0 h (left), 12 h (middle) and 72 h (right) across WT, Enhancer-KO-1 and Enhancer-KO-2 (three replicates per condition, Rep1-Rep3). Values in tiles denote expression (FPKM), and colors indicate row-wise Z-score-normalized expression.

Supplementary Figure 7

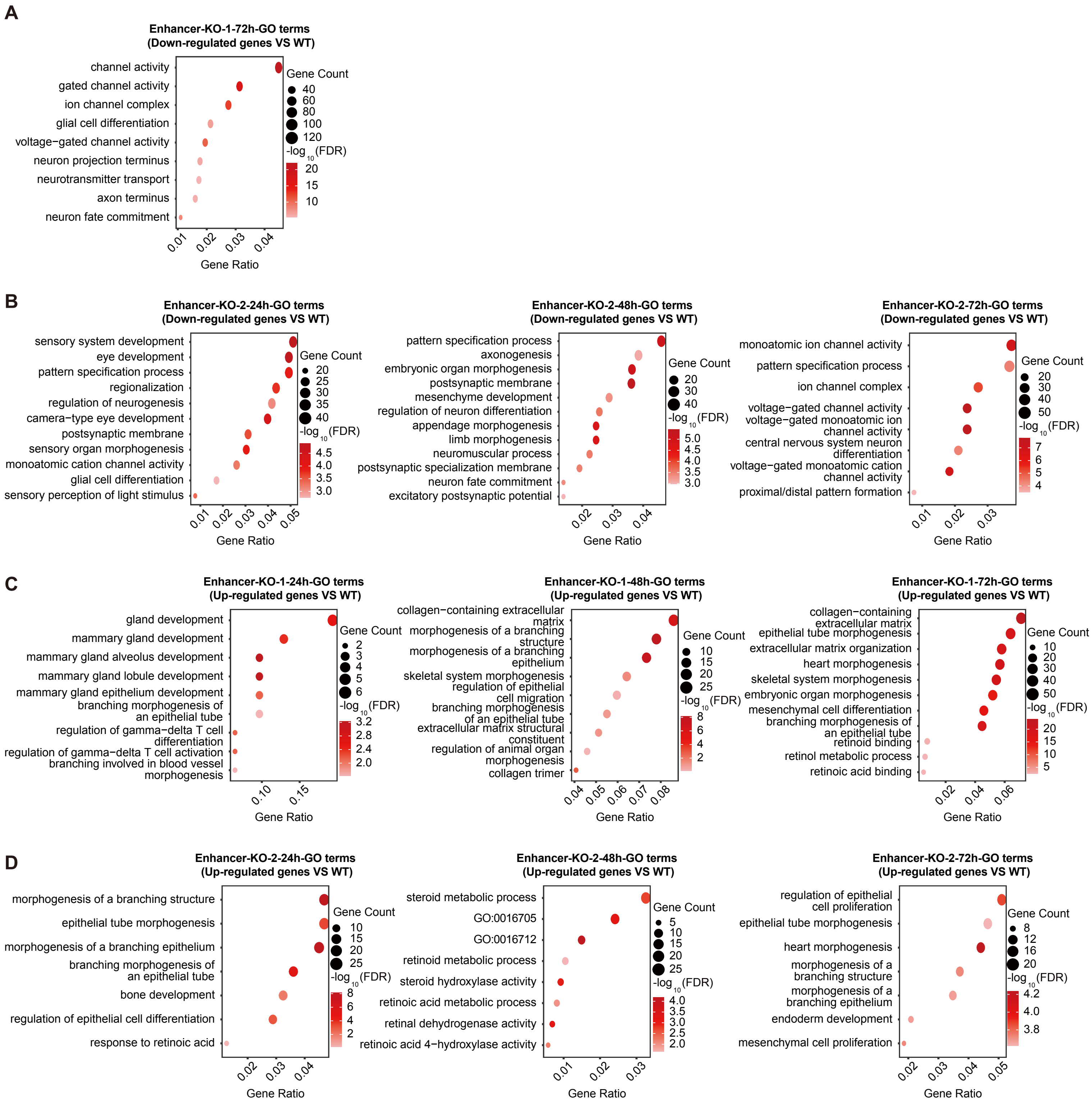

Supplementary Fig. 7.

GO enrichment analysis of Enhancer-KO DEGs across the RA time course

A: GO enrichment dot plot for downregulated genes in Enhancer-KO-1 relative to WT at 72 h after RA exposure. The x-axis indicates Gene Ratio. Dot size indicates Gene Count, and dot color indicates −log10(FDR) (legend at right).

B: GO enrichment dot plots for downregulated genes in Enhancer-KO-2 relative to WT at 24 h (left), 48 h (middle) and 72 h (right) after RA exposure.

C: GO enrichment dot plots for upregulated genes in Enhancer-KO-1 relative to WT at 24 h (left), 48 h (middle) and 72 h (right) after RA exposure.

D: GO enrichment dot plots for upregulated genes in Enhancer-KO-2 relative to WT at 24 h (left), 48 h (middle) and 72 h (right) after RA exposure.

Supplementary Figure 8

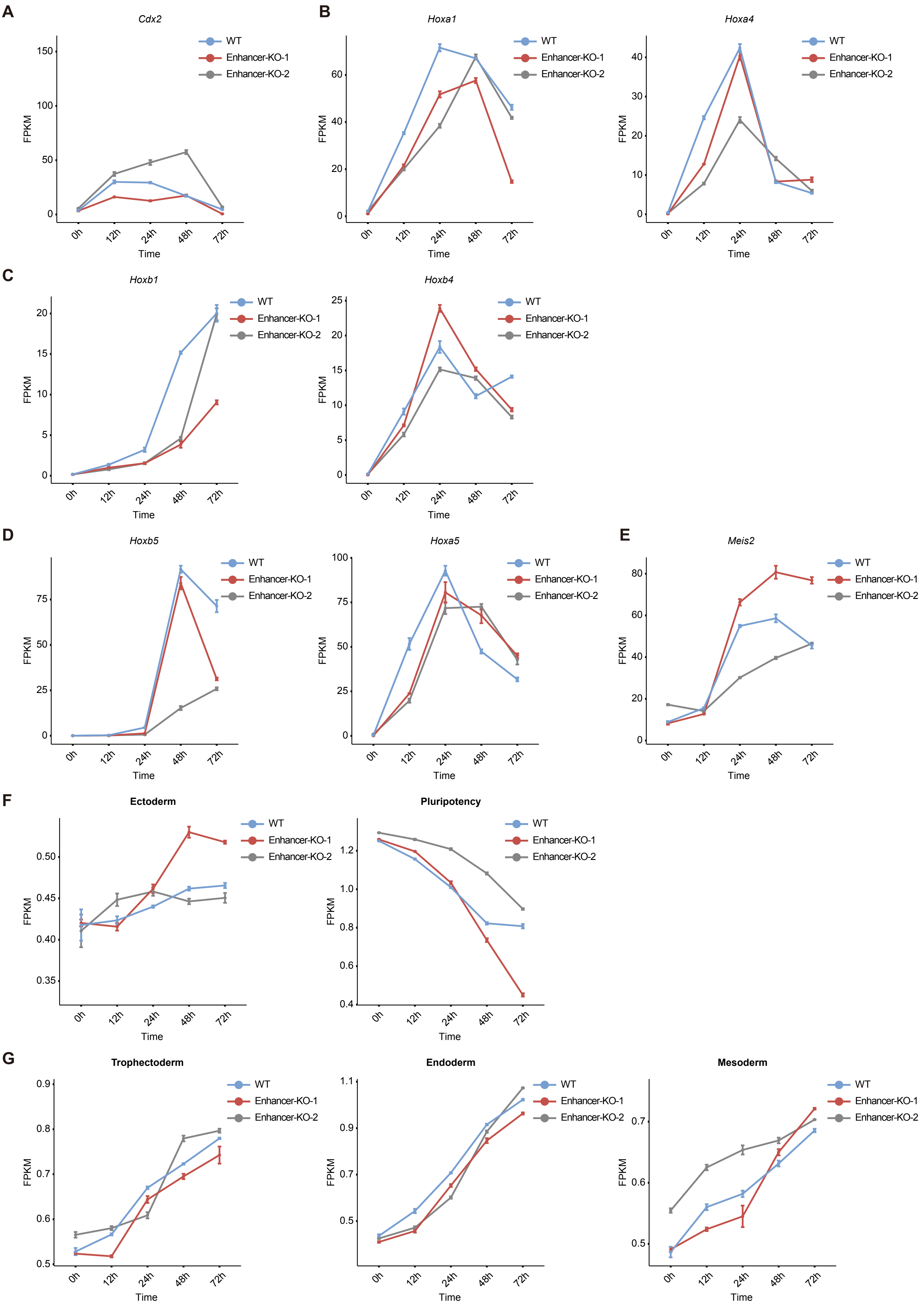

Supplementary Fig. 8.

Axial/Hox transcriptional trajectories and developmental-program dynamics in Enhancer-KO clones

A-E: Bulk RNA-seq-derived expression trajectories (FPKM) of selected axial-patterning and Hox-associated transcripts across the RA time course (0, 12, 24, 48 and 72 h) in WT and two independent Enhancer-KO clones (Enhancer-KO-1 and Enhancer-KO-2). Lines are color-coded by genotype (WT, blue; Enhancer-KO-1, red; Enhancer-KO-2, grey). (**A**) *Cdx2*. (**B**) *Hoxa1* and *Hoxa4*. (**C**) *Hoxb1* and *Hoxb4*. (**D**) *Hoxb5* and *Hoxa5*. (**E**) *Meis2*.

F-G: FPKM-based expression profiles of the indicated developmental programs across the RA time course (0, 12, 24, 48 and 72 h) in WT, Enhancer-KO-1 and Enhancer-KO-2. (**F**) Ectoderm and pluripotency programs. (**G**) Trophectoderm, endoderm and mesoderm programs.

Supplementary Figure 9

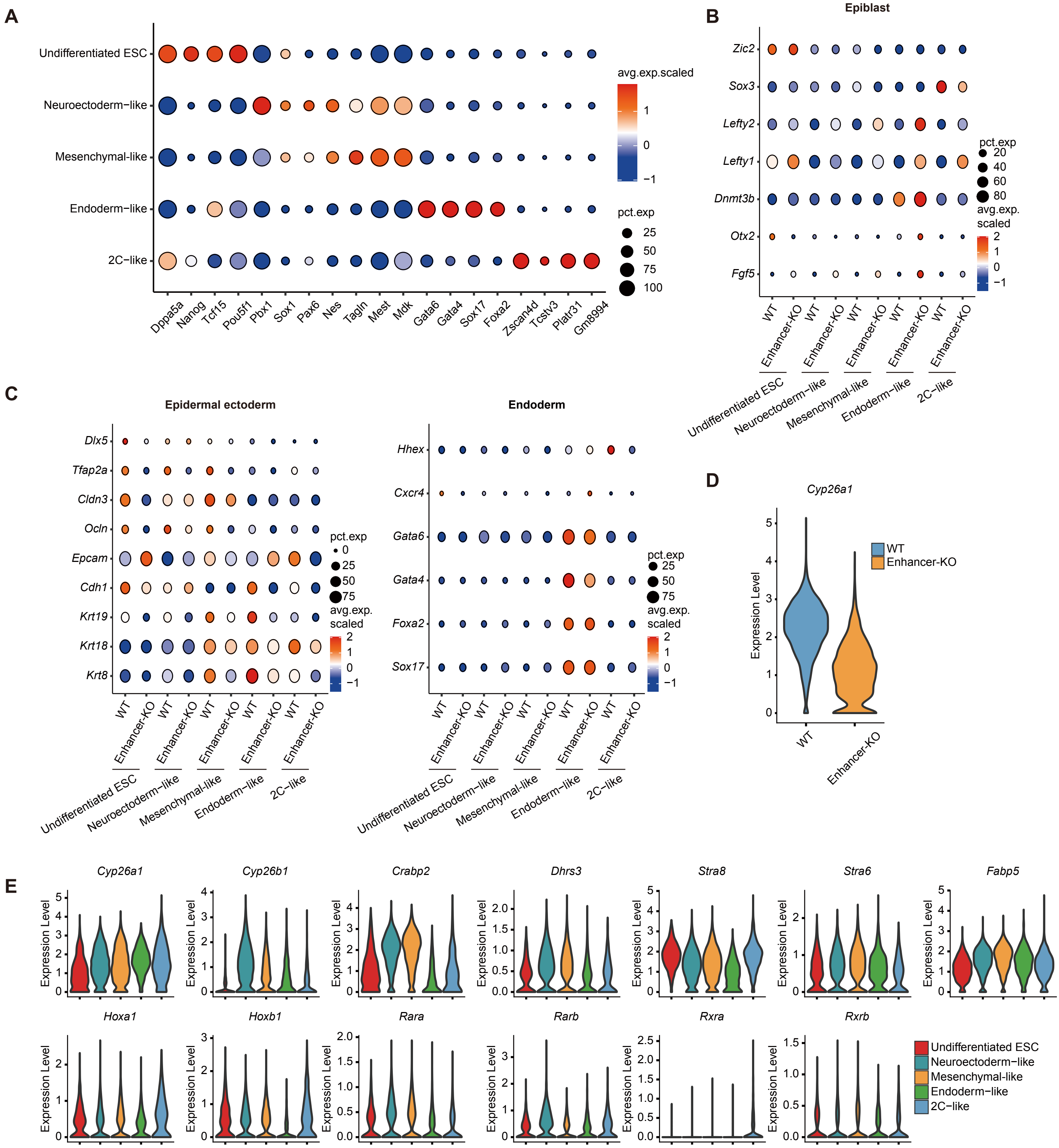

Supplementary Fig. 9.

State-resolved marker expression and RA-related gene expression in Enhancer-KO scRNA-seq profiles

A: Dot plot summarizing expression of representative state markers across annotated cell states (Undifferentiated ESC, Neuroectoderm-like, Mesenchymal-like, Endoderm-like and 2C-like). Dot size indicates the proportion of cells expressing each gene, and dot color indicates scaled average expression.

B: Dot plot of epiblast-associated markers shown for each annotated cell state, split by genotype (WT versus Enhancer-KO; paired columns as labelled).

C: Dot plots of epidermal ectoderm markers and endoderm markers shown for each annotated cell state, split by genotype (WT versus Enhancer-KO; paired columns as labelled).

D: Violin plot comparing *Cyp26a1* expression between WT and Enhancer-KO cells.

E: Violin plots showing expression distributions of genes involved in RA metabolism and handling, representative RA-responsive genes, and RA receptors across annotated cell states.

Supplementary Figure 10

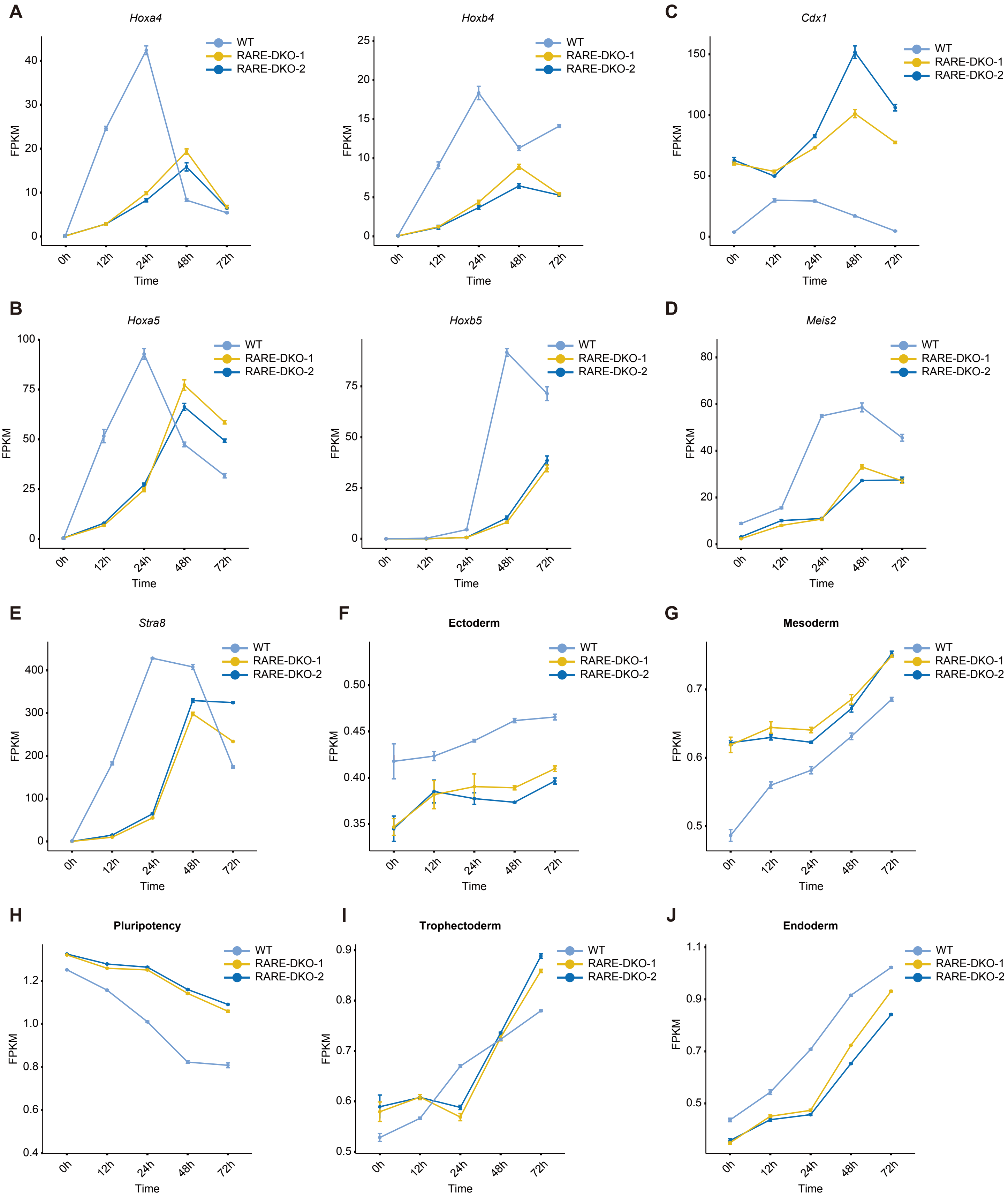

Supplementary Fig. 10.

Hox/RA-responsive gene trajectories and developmental-program dynamics in RARE-DKO cells

A-E: Bulk RNA-seq-derived expression trajectories (FPKM) across the RA time course (0, 12, 24, 48 and 72 h) for WT and two independent RARE-DKO lines (RARE-DKO-1 and RARE-DKO-2). (**A**) *Hoxa4* (left) and *Hoxb4* (right). (**B**) *Hoxa5* (left) and *Hoxb5* (right). (**C**) *Cdx1*. (**D**) *Meis2*. (**E**) *Stra8*.

F-J: FPKM-based expression profiles of the indicated developmental programs (0, 12, 24, 48 and 72 h) for WT, RARE-DKO-1 and RARE-DKO-2. (**F**) Ectoderm. (**G**) Mesoderm. (**H**) Pluripotency. (**I**) Trophectoderm. (**J**) Endoderm.

Supplementary Figure 11

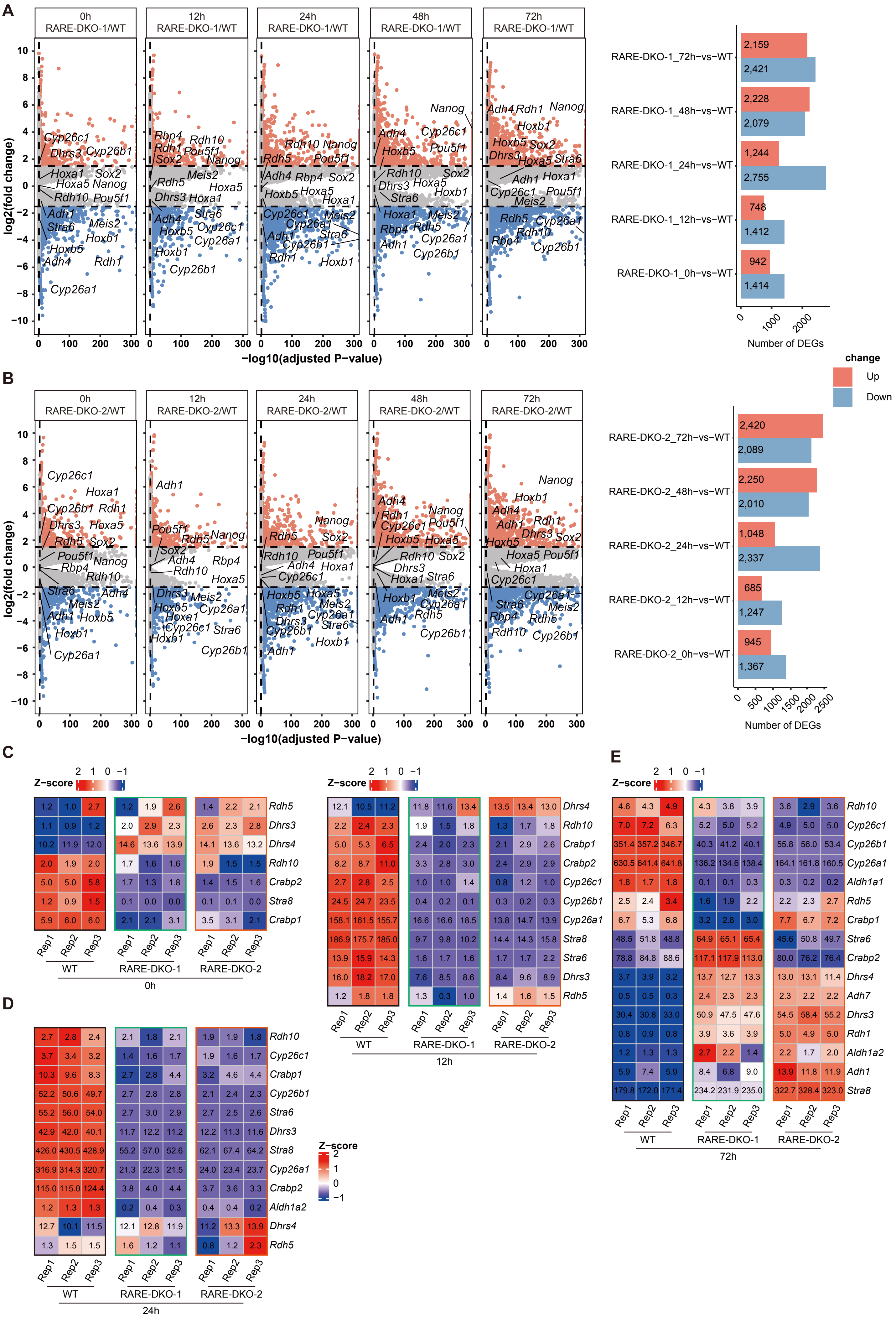

Supplementary Fig. 11.

Time-resolved differential expression and RA metabolism/handling gene expression in RARE-DKO cells

A: Volcano plots showing differential gene expression in RARE-DKO-1 relative to WT at 0, 12, 24, 48 and 72 h after RA exposure. The x-axis denotes −log10(adjusted P value) and the y-axis denotes log2(fold change). Right, horizontal bar plots summarizing the number of DEGs at each time point (Up, red; Down, blue), with counts indicated on the bars.

B: Volcano plots and DEG counts for RARE-DKO-2 relative to WT at 0, 12, 24, 48 and 72 h after RA exposure.

C: Heatmap of RA metabolism/handling transcripts at 0 and 12 h across WT, RARE-DKO-1 and RARE-DKO-2 (three replicates per genotype, Rep1-Rep3). Values in tiles indicate FPKM, and colors indicate row-wise Z-score-normalized expression.

D: Heatmap of RA metabolism/handling transcripts at 24 h across WT, RARE-DKO-1 and RARE-DKO-2 (three replicates per genotype, Rep1-Rep3). Values in tiles indicate FPKM, and colors indicate row-wise Z-score-normalized expression.

E: Heatmap of RA metabolism/handling transcripts at 72 h across WT, RARE-DKO-1 and RARE-DKO-2 (three replicates per genotype, Rep1-Rep3). Values in tiles indicate FPKM, and colors indicate row-wise Z-score-normalized expression.

Supplementary Figure 12

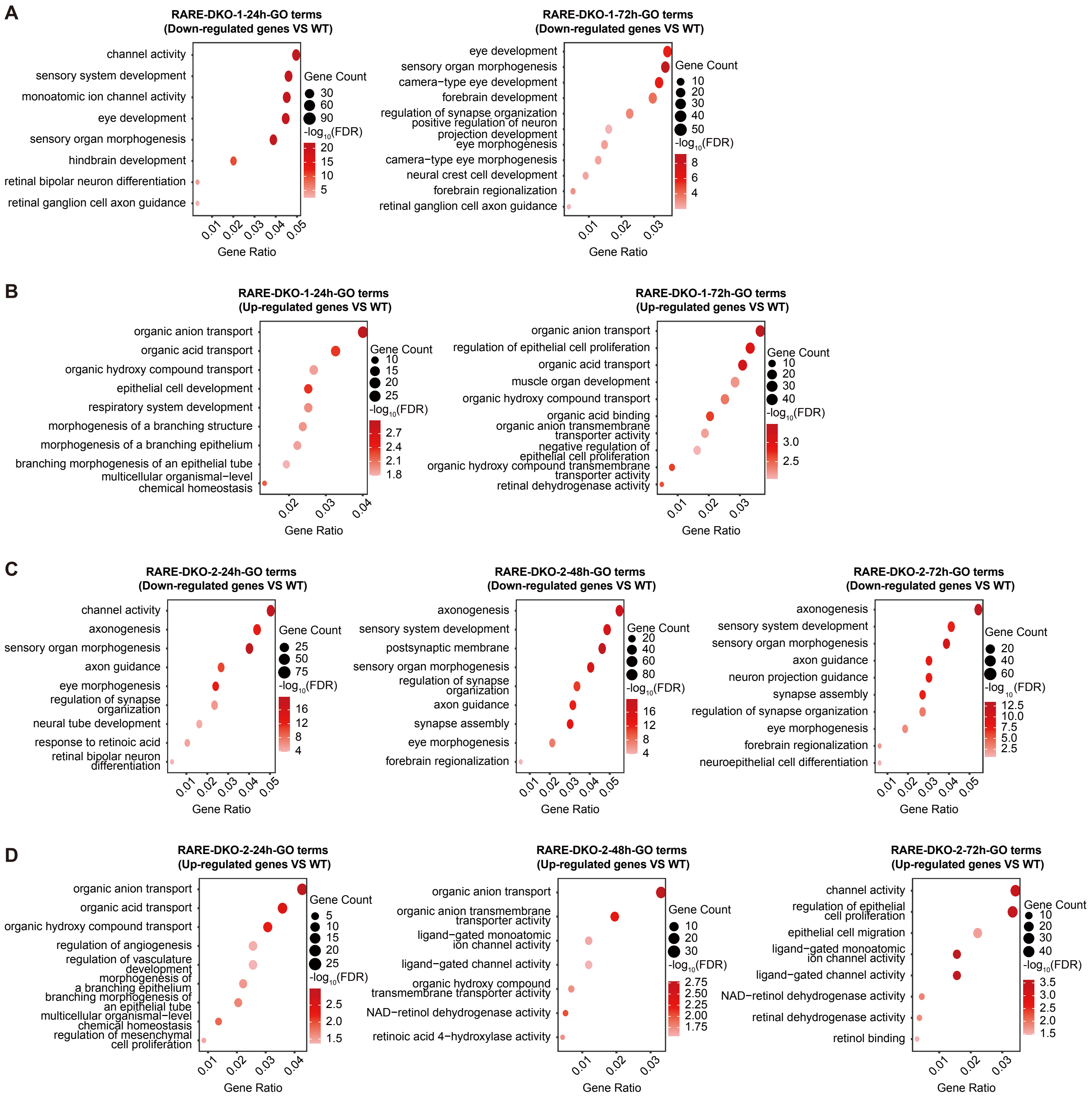

Supplementary Fig. 12.

GO enrichment analysis of RARE-DKO DEGs across the RA time course

A: GO enrichment dot plots for downregulated genes in RARE-DKO-1 relative to WT at 24 h (left) and 72 h (right) after RA exposure. The x-axis indicates Gene Ratio (the fraction of genes in the input DEG set annotated to a given GO term). Dot size indicates Gene Count, and dot color indicates −log10(FDR).

B: GO enrichment dot plots for upregulated genes in RARE-DKO-1 relative to WT at 24 h (left) and 72 h (right) after RA exposure.

C: GO enrichment dot plots for downregulated genes in RARE-DKO-2 relative to WT at 24 h (left), 48 h (middle) and 72 h (right) after RA exposure.

D: GO enrichment dot plots for upregulated genes in RARE-DKO-2 relative to WT at 24 h (left), 48 h (middle) and 72 h (right) after RA exposure.

Supplementary Figure 13

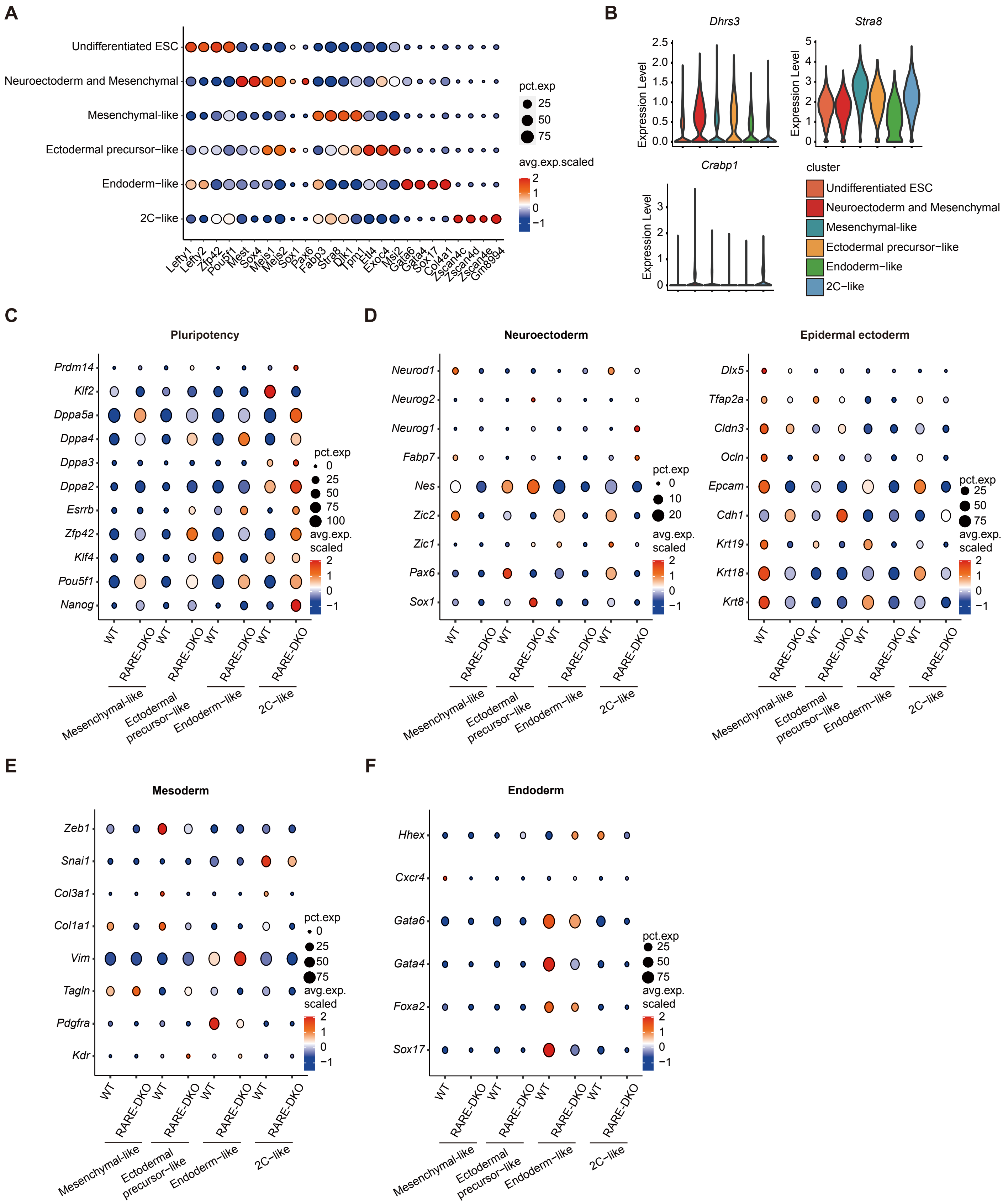

Supplementary Fig. 13.

Cell-state marker patterns and RA-related gene expression in RARE-DKO cells

A: Dot plot summarizing expression of selected marker genes across annotated cell states. Dot size indicates the fraction of cells expressing each gene (pct.exp), and dot color indicates scaled average expression (avg.exp.scaled).

B: Violin plots showing per-cell expression distributions of *Dhrs3* and *Stra8* (top) and *Crabp1* (bottom) across the annotated cell states.

C-F: Dot plots showing developmental-state-associated marker expression within different cell states, split by genotype (WT versus RARE-DKO). (**C**) pluripotency-associated markers. (**D**) neuroectoderm-associated markers (left) and epidermal ectoderm-associated markers (right). (**E**) mesoderm/mesenchymal-associated markers. (**F**) endoderm-associated markers.

Supplementary Figure 14

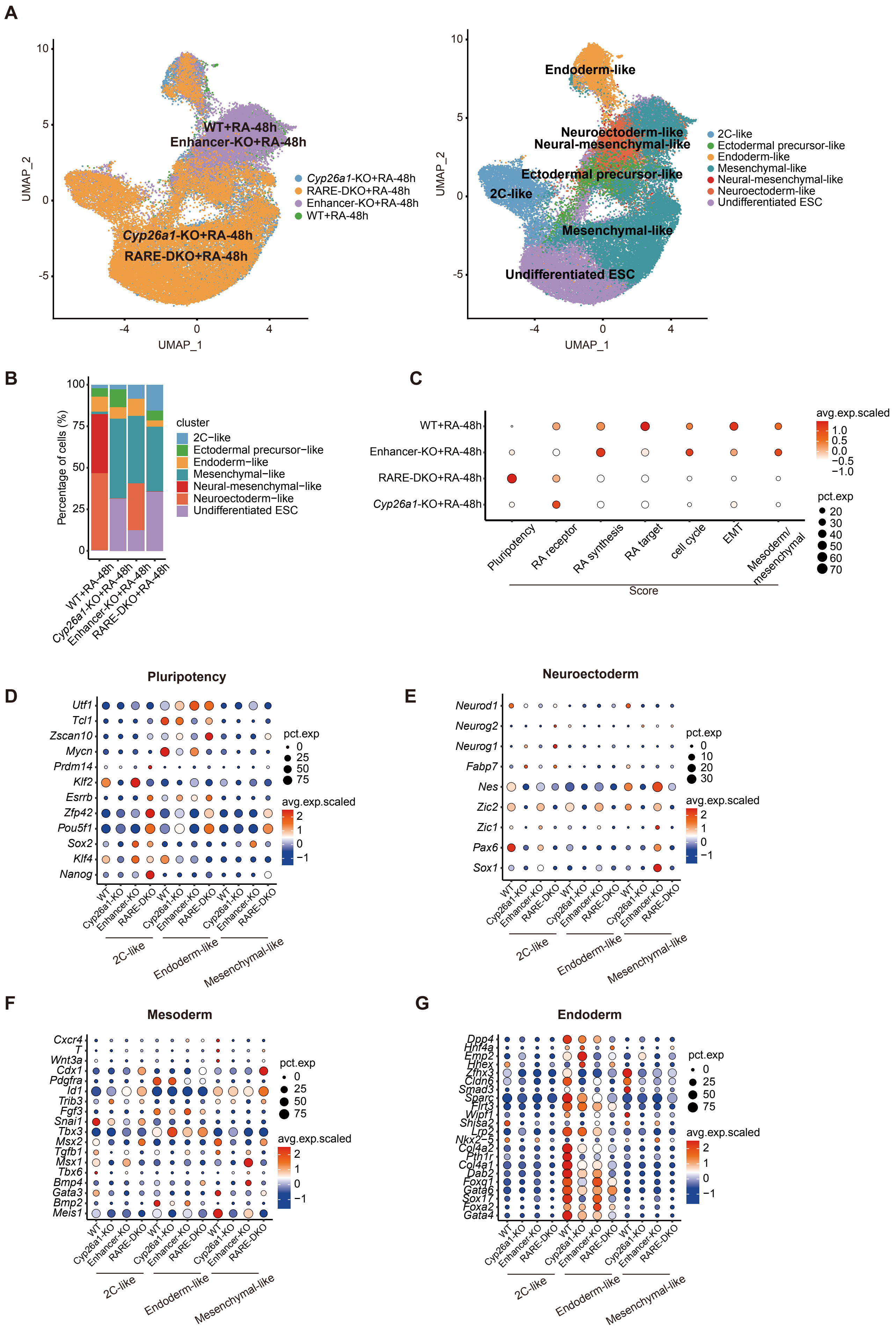

Supplementary Fig. 14.

Cross-genotype comparison of RA-induced differentiation states across the *Cyp26a1* regulatory axis

A: Integrated UMAP embedding of single-cell RNA-seq profiles from WT, *Cyp26a1*-KO, Enhancer-KO and RARE-DKO cells after 48 h of RA treatment. Left, cells are colored by genotype. Right, the same embedding is colored by annotated cell states.

B: Cell-state composition of WT, *Cyp26a1*-KO, Enhancer-KO and RARE-DKO samples after 48 h of RA treatment, shown as the percentage of cells assigned to each annotated state.

C: Dot plot comparing selected selected gene-set scores across WT, Enhancer-KO, RARE-DKO and *Cyp26a1*-KO cells. Dot color indicates scaled average score, and dot size indicates the proportion of cells contributing to each gene-set signal, as plotted.

D-G: Dot plots comparing developmental-state-associated marker expression among WT, *Cyp26a1*-KO, Enhancer-KO and RARE-DKO cells within the indicated cell states. Dot size indicates the proportion of cells expressing each gene, and dot color indicates scaled average expression. (D) Pluripotency-associated markers. (E) Neuroectoderm-associated markers. (F) Mesoderm-associated markers. (G) Endoderm-associated markers.

Supplementary Figure 15

A

Pan-cancer analysis of CYP26A1

dataset	tumor_median	normal_median	log2fc	p-value
LUSC	1.18	0.14	1.62	2.87e-52
UCS	1.19	0.17	1.28	2.39e-13
TGCT	0.79	0.61	1.11	4.37e-12
PCPG	0.68	0.18	1.03	0.255
ESCA	0.2	0	0.86	5.17e-19
PAAD	0.48	0.03	0.79	1.82e-24
UCEC	0.37	0.16	0.48	2.48e-06
HNSC	0.24	0.27	0.44	0.0839
SKCM	0.19	0.11	0.43	1.39e-23
CESC	0.26	0.28	0.41	0.176
OV	0.34	0.12	0.38	3.66e-08
SARC	0.11	0	0.38	4.6e-14
READ	0.14	0	0.31	9.84e-08
LUAD	0.19	0.14	0.27	1.33e-11
BLCA	0.12	0.04	0.25	0.0834
BRCA	0.78	0.32	0.24	0.000287
KICH	0.1	0.1	0.24	0.00405
COAD	0.12	0.06	0.19	4.52e-16
THCA	0.15	0.1	0.17	5.7e-11
THYM	0.19	0.65	0.15	0.872
STAD	0.08	0	0.13	2.32e-12
**KIRC**	**0.11**	**0.07**	**0.11**	**0.00353**
PRAD	0.07	0.09	0.05	0.221
KIRP	0.04	0.1	-0.01	0.915
ACC	0.06	0.08	-0.07	0.0606
LGG	0.32	0.41	-0.14	0.000192
GBM	0.38	0.99	-0.42	6.71e-08
LIHC	0.36	2.58	-1.87	9.73e-53
LAML	0.07	2.28	-2.11	3.82e-126
CHOL	0.18	4.1	-2.77	8.25e-11

B

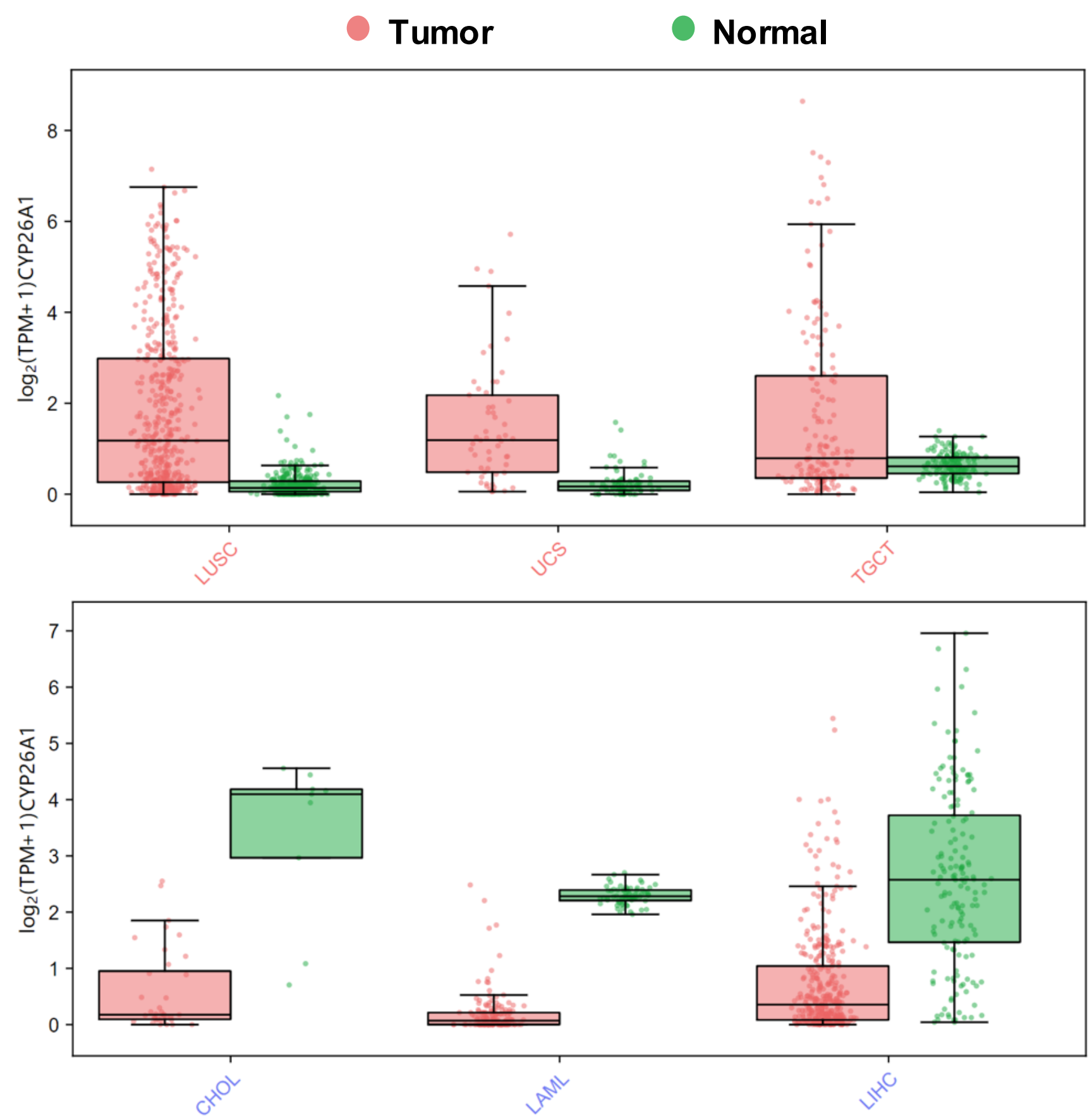

C

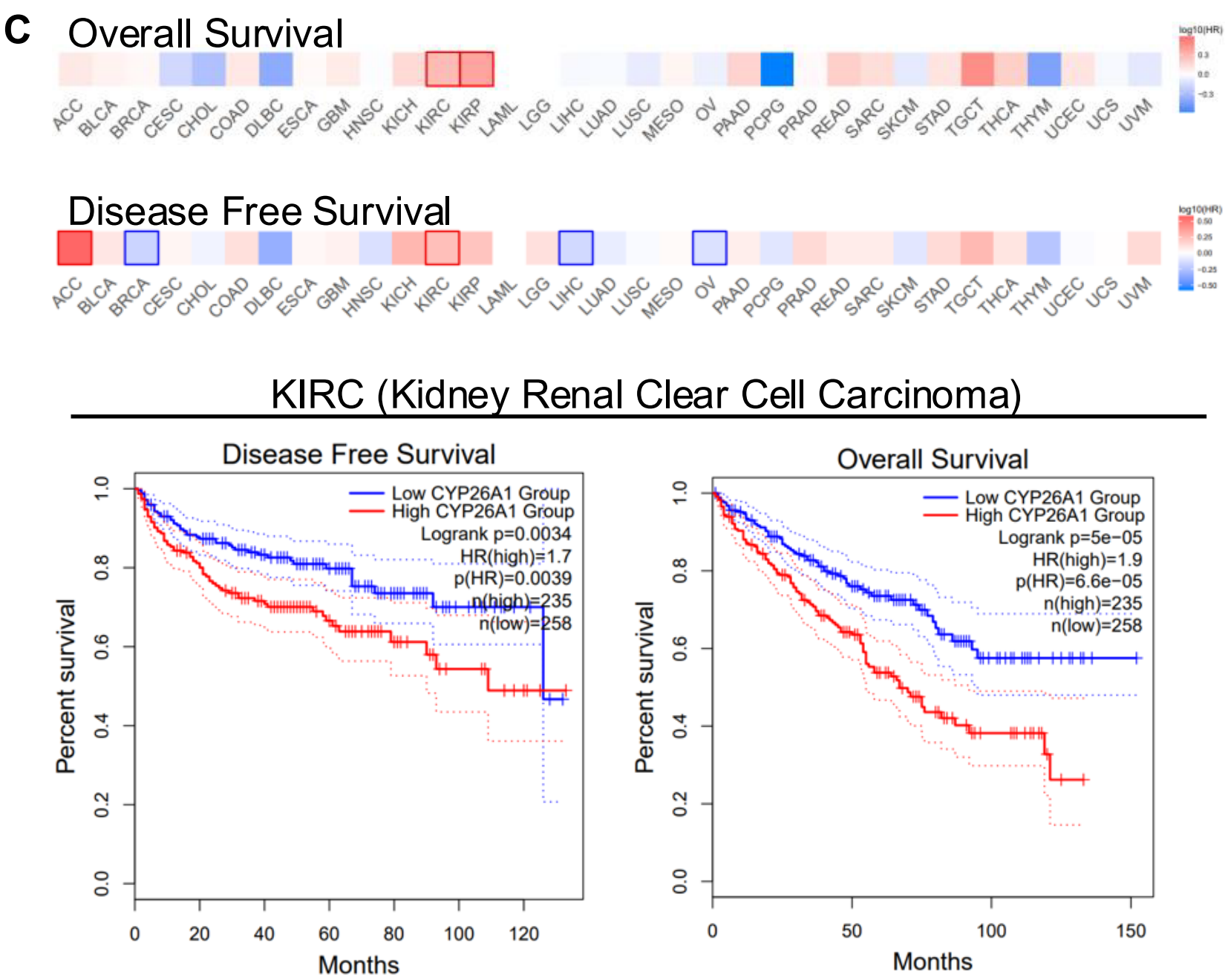

D

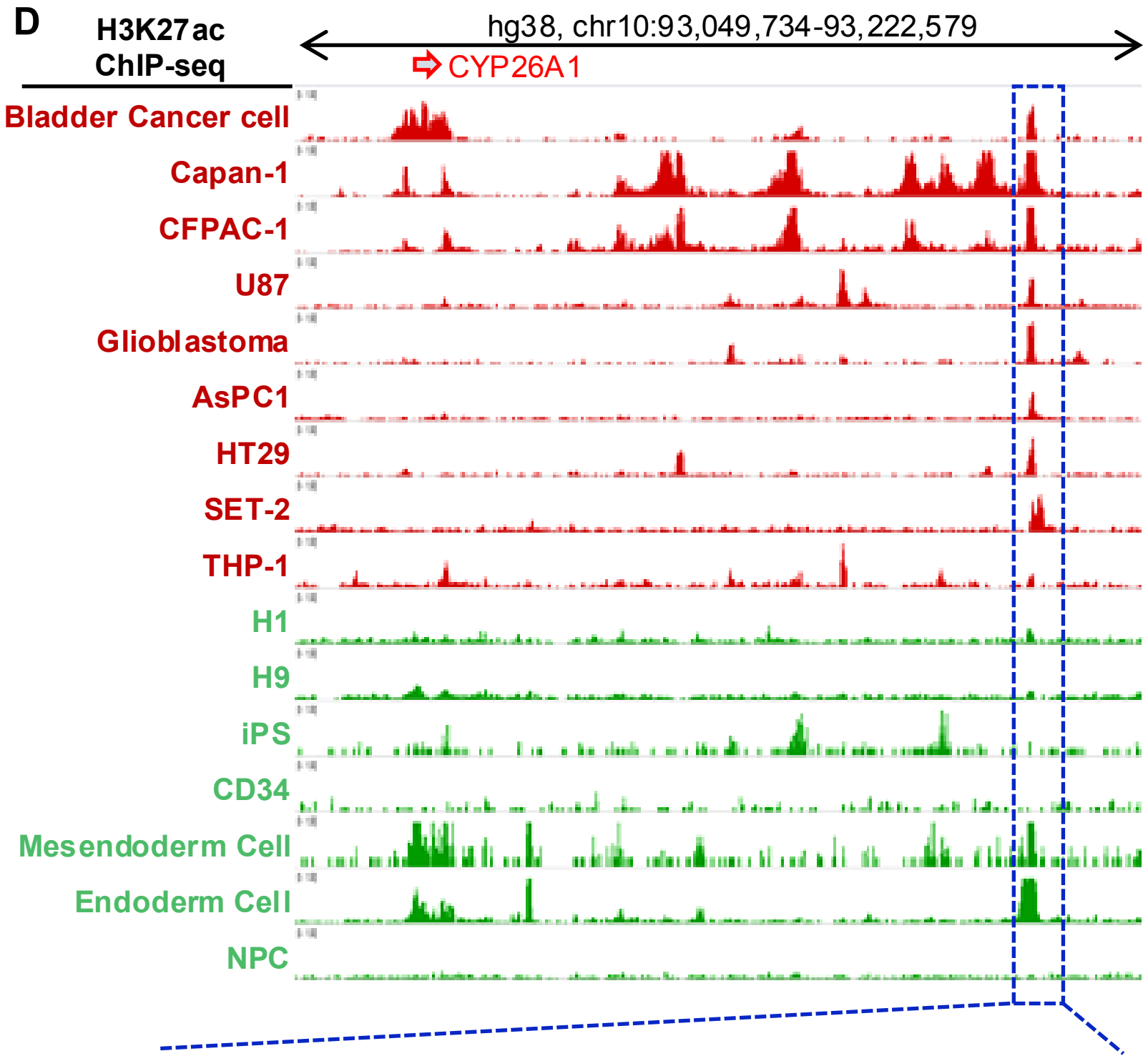

5'-CAGGAAGTGTCTC**AGTTCACGGAAAGGGCA**GCGAGGATATA-3'

Model ID	Model name	Score	Relative score	Strand	predicted site sequence
MA0159.1	RARA::RXRA	17.337	0.934001015542404	1	AGTTCACGGAAAGGGCA

E

RARA::RXRA from the JASPAR CORE database

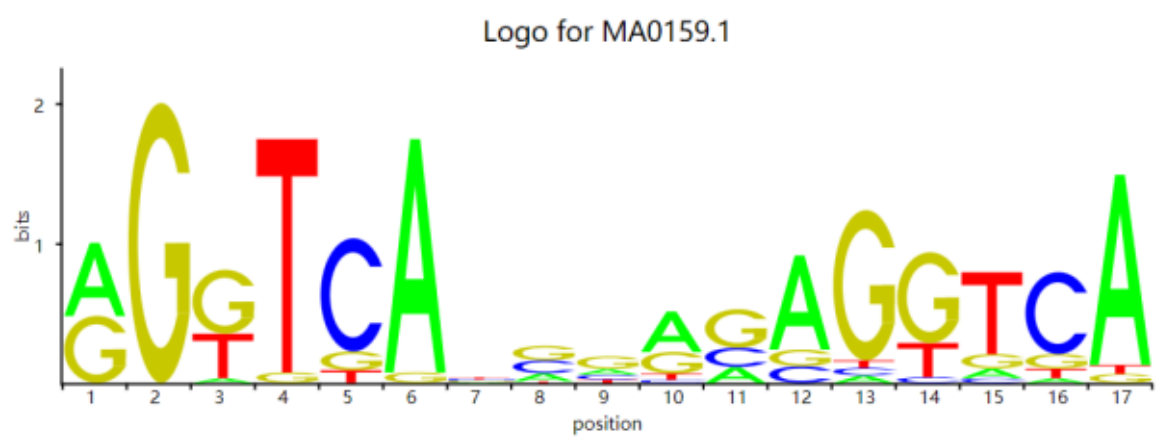

Supplementary Fig. 15.

Pan-cancer expression, prognostic associations, and epigenomic features of CYP26A1

A: Summary of CYP26A1 differential expression across multiple cancer types. The table presents tumor median expression, normal median expression, log2 fold change (log2FC), and P values for CYP26A1.

B: Representative boxplots comparing CYP26A1 expression between tumor and normal tissues in selected cancer types. The top panel displays tumor types with significantly upregulated CYP26A1 expression (LUSC, UCS, and TGCT), while the bottom panel shows tumor types with downregulated expression (CHOL, LAML, and LIHC). Data are presented as log2(TPM+1).

C: Association of CYP26A1 expression with patient survival. Upper panels show pan-cancer heatmaps for overall survival (OS) and disease-free survival (DFS). Lower panels present Kaplan-Meier survival curves for kidney renal clear cell carcinoma (KIRC), stratified by CYP26A1 expression, with log-rank P values and hazard ratios (HRs) as indicated.

D: Epigenetic signal tracks at the CYP26A1 genomic locus. H3K27ac ChIP-seq profiles are shown for human cancer cell lines (red) and pluripotent or differentiated human cell types (green) across the hg38 region chr10:93,049,734-93,222,579. The highlighted box indicates a s shared H3K27ac-enriched region containing a putative enhancer element the sequence 5'-CAGGAAGTGTCTCAGTTCACGGAAAGGGCAGCGAGGATATA -3'.

E: Prediction of transcription factor binding sites. The RARA/RXRA binding motif logo (Model ID: MA0159.1) and the predicted binding site sequence (AGTTCACGGAAGGGCA) within the putative enhancer region are shown.

The data analyses in (A-C) were performed using the GEPIA2 and GEPIA3 web servers (http://gepia2.cancer-pku.cn/#index and https://gepia3.bioinfoliu.com/). The transcription factor binding site prediction in (E) was conducted utilizing the JASPAR CORE database (http://jaspar2016.genereg.net/).

Supplementary Figure 16

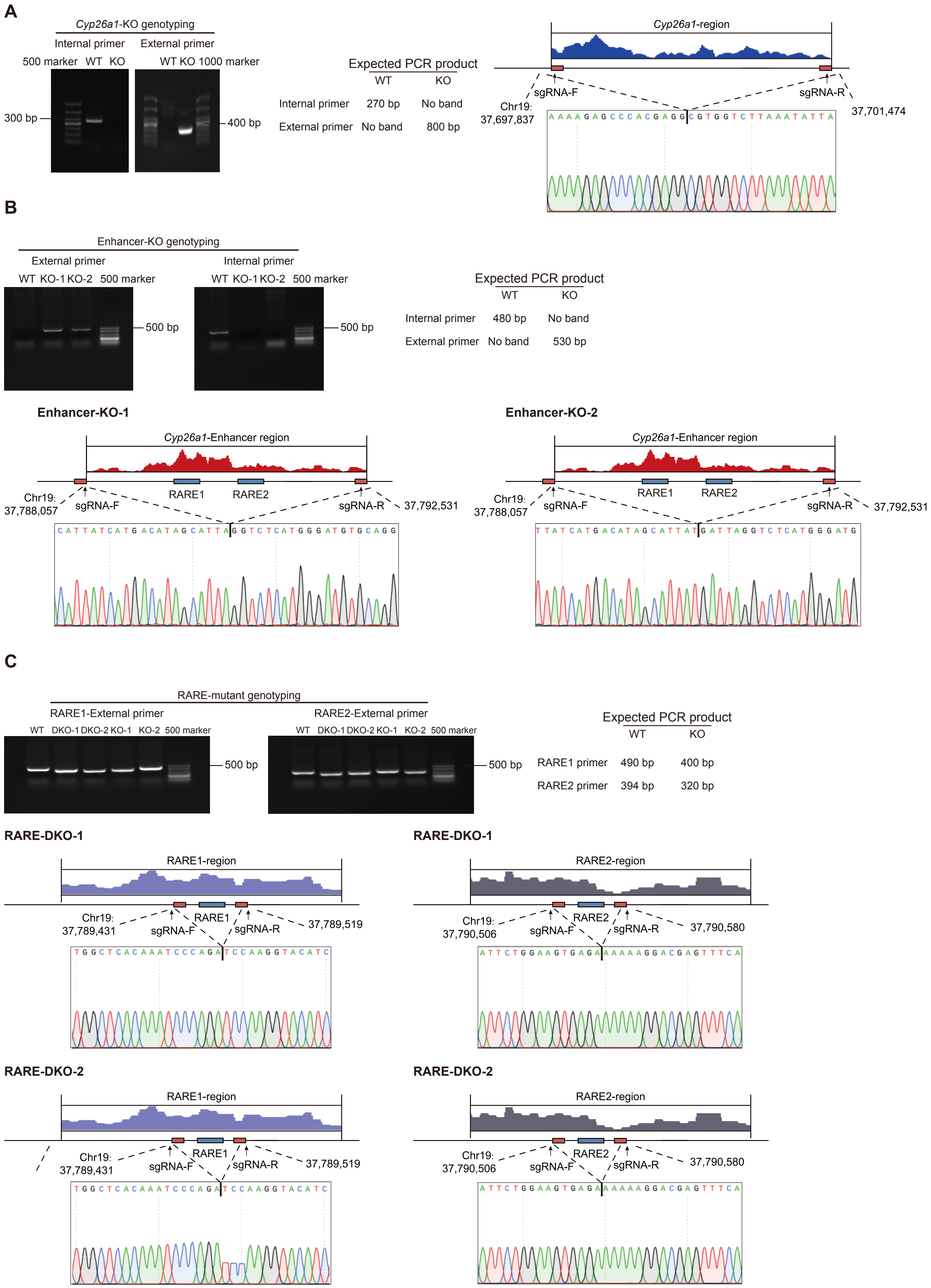

Supplementary Fig. 16.

Genotyping and sequence validation of *Cyp26a1*, distal enhancer, and RARE knockout mESC lines

A: gDNA-PCR genotyping of WT and *Cyp26a1*-KO cells using internal and external primer pairs. DNA markers are shown as indicated. Right, schematic of the edited *Cyp26a1* locus and representative Sanger sequencing chromatogram confirming the deletion junction.

B: gDNA-PCR genotyping of WT, Enhancer-KO-1 and Enhancer-KO-2 cells using external and internal primer pairs. Representative Sanger sequencing chromatograms of the deletion junctions are shown for Enhancer-KO-1 and Enhancer-KO-2.

C: gDNA-PCR genotyping of RARE1 and RARE2 regions in WT, RARE-DKO-1, RARE-DKO-2, RARE1-KO and RARE2-KO cells, as indicated. Representative locus schematics and Sanger sequencing chromatograms confirm editing of RARE1 and RARE2 in the two RARE-DKO clones.
